# Advancing breast cancer treatment through dual targeting CAR T cell therapy

**DOI:** 10.1007/s12672-025-04195-3

**Published:** 2025-12-05

**Authors:** Mujibullah Sheikh, Dilip Madia, Umesh B. Telrandhe, Harpritkaur Bagga, Arya Deshmukh

**Affiliations:** 1 Department of pharmaceutics, Datta Meghe College of Pharmacy DMIHER (Deemed to be University), Wardha, 442001 Maharashtra India; 2 Tulaskar College of Pharmacy Hinganghat Dist, Wardha, 442301 M.S India

**Keywords:** Breast cancer, Dual-targeting CAR-T-cell therapy, Tumor antigen heterogeneity, Tumor microenvironment, Tandem CAR constructs.

## Abstract

Dual targeting chimeric antigen receptor (CAR) T cell therapy offers a next generation strategy to overcome the limitations of single antigen approaches in breast cancer, including antigen heterogeneity, immune escape, and the suppressive tumor microenvironment. This review summarizes current advances in vector design, comparing lentiviral, retroviral, and transposon platforms, and highlights key manufacturing challenges such as reduced transduction efficiency in dual scFv constructs (65–75% versus 92–98% for single target CAR T cells), prolonged vein to vein timelines (18–28 days), and increased production costs ($500,000–$700,000 per treatment). Safety considerations including cytokine release syndrome, neurotoxicity, and on target off tumor effects are discussed alongside the lack of predictive biomarkers for patient selection. The review further explores innovations in CAR engineering such as tandem and bicistronic configurations, logic gated SynNotch circuits, and affinity tuned costimulatory domains designed to enhance persistence and specificity. Advances in automated bioreactor systems, nonviral delivery methods, and AI guided manufacturing have improved scalability and cost efficiency. Integration with checkpoint inhibitors, oncolytic viruses, and stromal remodeling agents shows promise for overcoming tumor microenvironmental barriers, while single cell antigen profiling and circulating tumor DNA monitoring enable more personalized dual antigen targeting. Emerging frontiers such as in vivo CAR engineering provide additional opportunities to simplify manufacturing and expand accessibility. Finally, regulatory innovation, decentralized production, and value based reimbursement models are discussed as essential components for translating dual targeting CAR T cell therapy from experimental development to a clinically viable and economically sustainable treatment for breast cancer.

**Graphical abstract:**

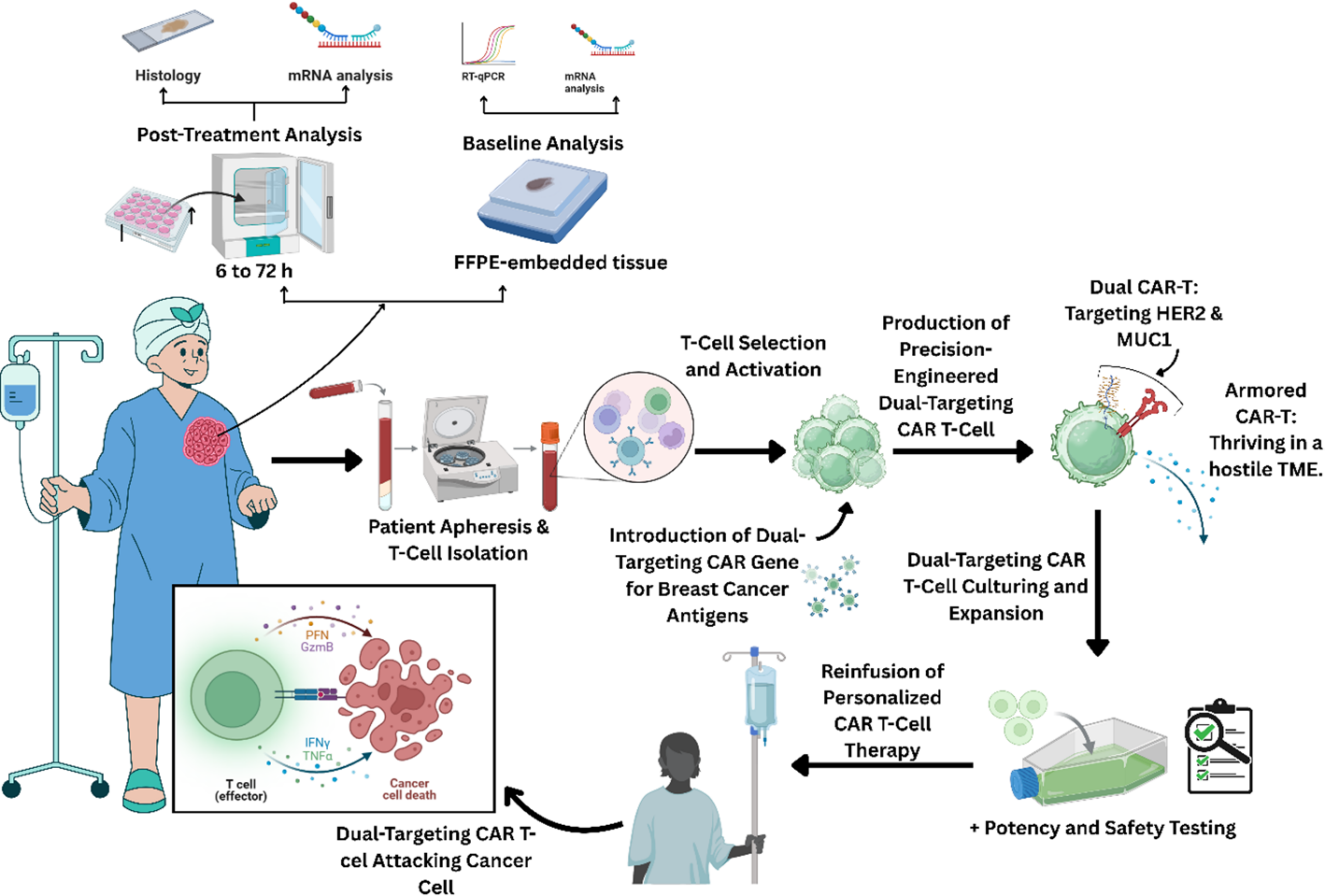

## Introduction

**Fig. 1:**
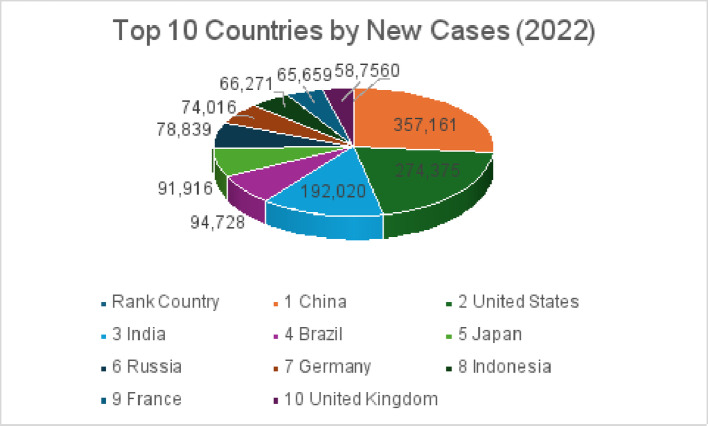
Age standardized incidence rate per 100,000 population plotted against the total number of new cancer cases in 2022 for the ten countries with the highest case counts, based on data reported in source [1]

**Fig. 2:**
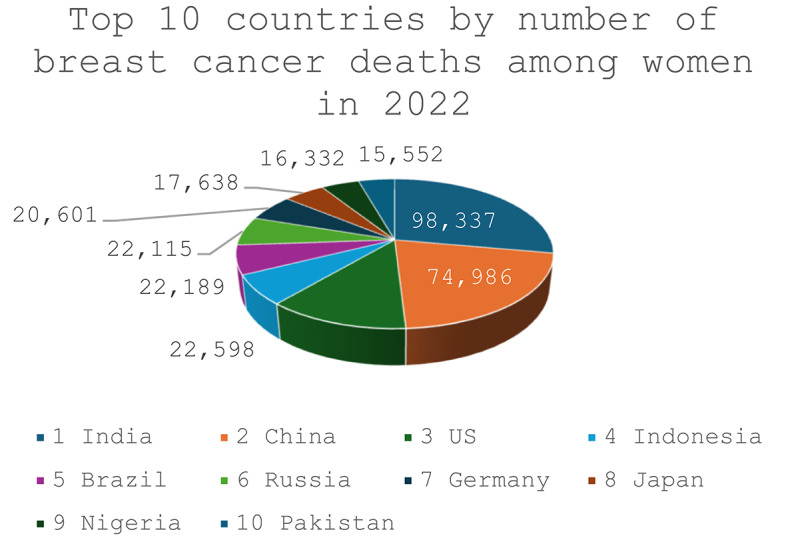
Data for the ten countries with the highest number of breast cancer deaths among women in 2022 were extracted from source [1]

Breast cancer remains the most commonly diagnosed cancer among women worldwide, with striking disparities in incidence and mortality across nations. In 2022, China reported the highest number of new breast cancer cases (357,161), followed by the United States (274,375) and India (192,020) (Fig. 1). Despite lower incidence, India experienced the greatest mortality (98,337 deaths), compared with 74,986 in China and 42,900 in the United States (Fig. 2), underscoring the unequal global burden of the disease [1]. Other nations, including Brazil, Russia, Germany, and Indonesia, also ranked among the top ten for both new cases and deaths, highlighting breast cancer’s widespread impact.

The heterogeneity of breast cancer classified into luminal A/B, HER2-positive, and triple-negative subtypes—presents major therapeutic challenges, particularly for aggressive forms that resist conventional chemotherapy, endocrine therapy, and HER2-targeted agents [2, 3]. Immunotherapy has emerged as a promising strategy, with immune checkpoint inhibitors (ICIs) targeting PD-1/PD-L1 signaling to reactivate T-cell function. However, clinical responses in metastatic breast cancer remain modest (15–20%) and largely restricted to PD-L1-positive triple-negative breast cancer (TNBC) [4, 5]. 

This limited success has shifted attention to CAR-T-cell therapy, an approach that has transformed the treatment of hematologic malignancies by achieving over 90% remission rates in CD19-positive leukemia [6]. Yet, despite encouraging preclinical results in breast cancer models targeting HER2, MUC1, or mesothelin [7–9], single-target CAR-T cells have demonstrated minimal clinical efficacy in solid tumors, including breast cancer, with objective response rates below 13% [10]. The central barriers are now well-recognized:

- **Antigen heterogeneity and immune escape**, where tumor cells downregulate or lose the targeted antigen (e.g., HER2), allowing them to evade recognition and destruction [10, 11].
- **The immunosuppressive tumor microenvironment (TME)**, marked by hypoxia, metabolic stress, regulatory T cells, and inhibitory molecules such as PD-L1 and TGF-β, which collectively impair CAR-T-cell activation and persistence [10].
- **Toxicity and off-target effects**, including cytokine release syndrome (CRS) and damage to normal tissues expressing low levels of the targeted antigen [6, 12].

Unlike single target CAR T cells, which remain vulnerable to antigen loss and have shown limited activity in solid tumors, dual targeting designs can recognize two tumor associated antigens simultaneously, reducing immune escape and improving tumor specificity. This approach also contrasts with immune checkpoint inhibitors, where clinical responses in metastatic breast cancer are modest and largely restricted to PD L1 positive triple negative disease Li et al., [154]. Monoclonal antibodies and antibody drug conjugates demonstrate targeted cytotoxicity, but their effectiveness is limited by antigen heterogeneity and acquired resistance. By integrating antigen level recognition with sustained T cell mediated cytotoxicity, dual target CAR T cells offer a mechanistically distinct immunotherapeutic strategy with the potential for more durable responses than single agent immunotherapies Zhang et al., [178].

These limitations define the critical failure of single-target CAR-T-cell therapy in breast cancer and form the rationale for the development of next-generation strategies. Emerging solutions, such as dual-target CAR-T cells that simultaneously recognize two tumor-associated antigens (e.g., HER2/MUC1 or B7-H3/CD276), aim to counter antigen escape, enhance specificity, and reduce off-tumor toxicity [13, 14]. Additionally, combining CAR-T cells with agents that remodel the TME such as TGF-β inhibitors [15], oncolytic viruses [16] or nanoparticle-delivered immunomodulators [16] further strengthens their therapeutic potential. Personalized dual-targeting approaches guided by patient-specific antigen profiles align closely with the principles of precision oncology and may offer a viable path to overcoming breast cancer heterogeneity [8].

## Overview of CAR-T-cell therapy technology

**Fig. 3:**
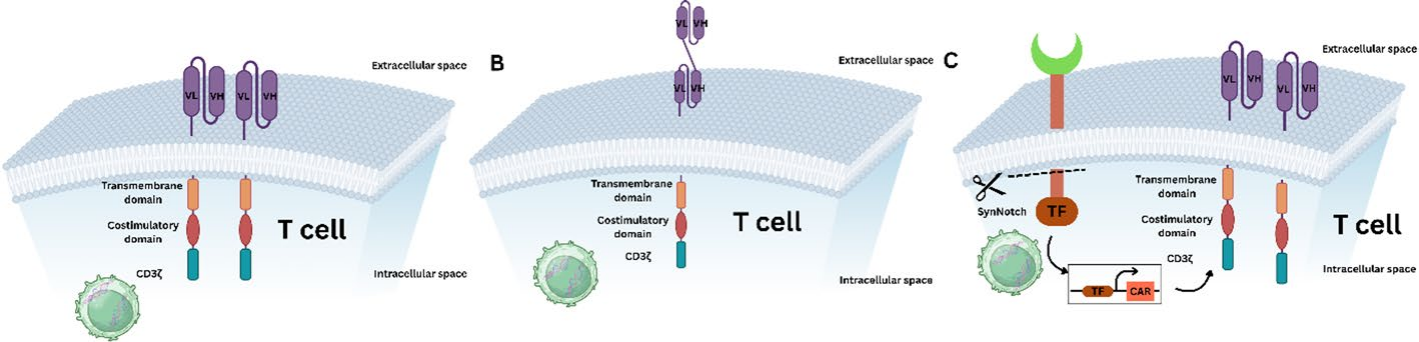
Schematic overview of bispecific CAR T-cell formats. ** A** Tandem dual CAR design that incorporates two antigen-binding domains in a single receptor. ** B** Parallel dual CAR format in which two independent CAR constructs are expressed on the same T cell. ** C** synNotch-inducible bispecific CAR system

The fundamental architecture of the CAR T-cell mechanism involves a combination of a unique set of protein domains that can target cancer cells with the objective of tumor-specific recognition and activation of T cells. The extracellular antigen-binding domain is composed of a single-chain variable fragment (scFv), which is produced most often as part of monoclonal antibodies and determines the target (e.g., CD19 in B-cell malignancies) [13, 17, 18]. This scFv is then attached to the hinge region, which is a spacer that supports antigen interaction through providing steric flexibility. Hinge length and composition (i.e., CD8alpha- or IgG-derived) play essential roles in binding kinetics and synapse formation [19, 20]. The CAR is anchored through its transmembrane domain (e.g., CD8α- or IgG-derived) to cross the lipid bilayer to interact with the intracellular signaling components [21]. More importantly, costimulatory domains (e.g., CD28 or CD8α) are incorporated with the CD3ζ immunoreceptor tyrosine-based activation motif (ITAM) aka CD3 to provide synergistic signals for T-cell proliferation, persistence and cytokine build-up [22]. Figure 3 illustrates three key strategies for engineering bispecific CAR-T cells, including the coexpression of two distinct CARs, tandem CAR constructs (tanCARs), and logic-gated SynNotch systems for sequential antigen targeting.

**Fig. 4:**
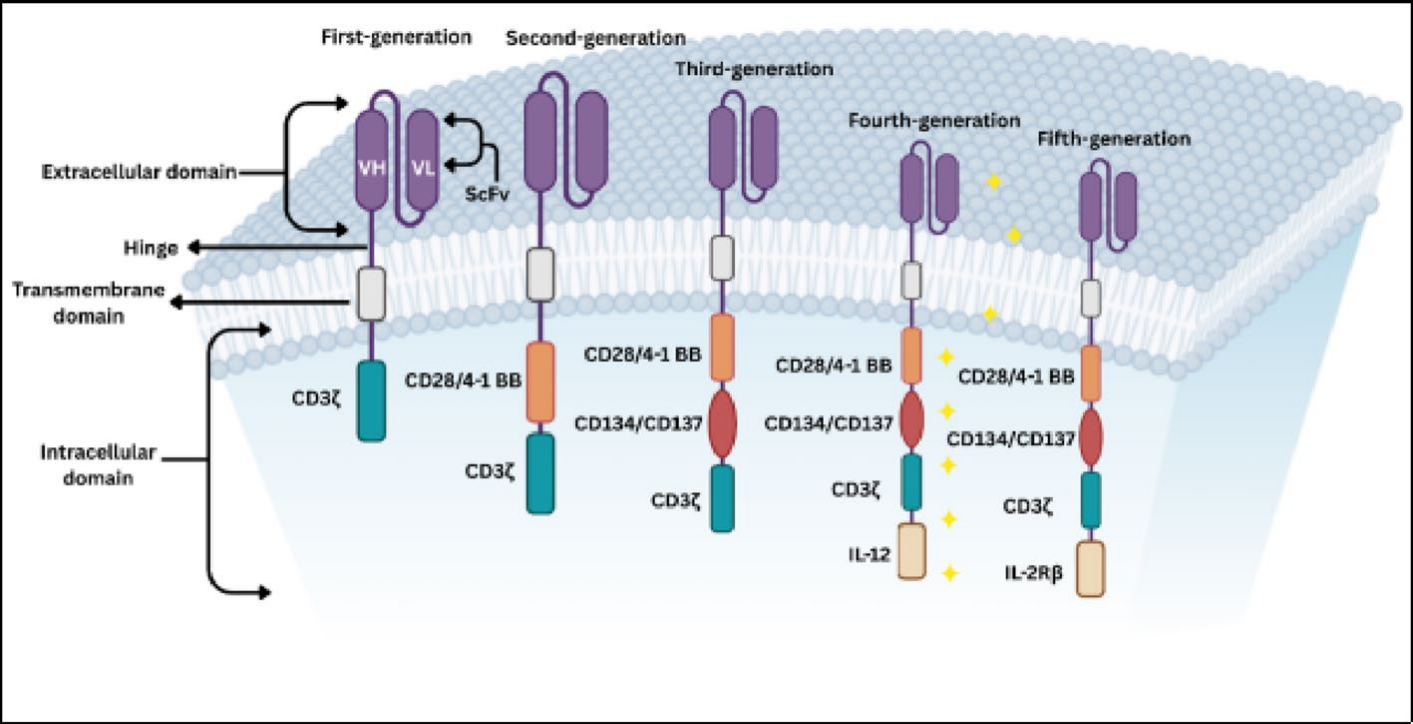
Structural evolution of the five generations of chimeric antigen receptors (CARs)

The evolution of CAR generations, as depicted in Fig. 4, reflects iterative optimizations of these domains:

- 1 st generation: CD3ζ alone → Limited persistence/effector function [17].
- 2nd Generation: Single costimulatory domain (CD28 or 4-1BB) → Enhanced survival/efficacy in B-cell malignancies [13].
- 3rd generation: Dual costimulatory domains (e.g., CD28 + 4-1BB) → amplified signaling but increased exhaustion risk [23].
- 4th/5th generations: “Armored” CARs with cytokine secretion (e.g., IL-12) or inducible safety switches [17, 23].

### Evolution from single to dual targeting CAR-T-cell constructs

The evolution from single- to dual-targeting CAR-T-cell constructs represents a new paradigm of adoptive cell therapy development: the intrinsic therapeutic limitation of the single-targeting therapy approach in hematological and solid tumors has become apparent. Although they are revolutionary in treating CD19 + malignancies, single-target CAR-T cells are associated with several setbacks, such as antigen escape (occurring in 30–60% of B-ALL patients [24]), tumor heterogeneity, and on-target/off-tumor toxicity [25]. Dual-targeting approaches using cotransduced CARs or tandem CARs (TanCAR) or bicistronic constructs represent a synergistic approach to recognize more efficiently but reduce escape mechanisms. For example, the concurrent downregulation of CCR9 and CD1a decreases fratricide and spares healthy T cells in T-ALL, eradicating malignant clones [26, 27]. Similarly, in hepatocellular carcinoma models, GPC3-directed CARs combined with AFP-specific TCRs address intratumor heterogeneity [28]. In acute myeloid leukemia (AML), the expression of the ADGRE2 antigen and CLEC12A antigen is differentially high and low, respectively. By exploiting this difference in antigen density, a dual-targeting mechanism optimizes signal integration: the ADGRE2-CAR can decrease ADGRE2 expression by coengaging CLEC12A, achieving more than 80% cytotoxicity in AML xenografts while preserving healthy cells [29].

Costimulatory domain selection further refines functionality. CD28 enhances initial activation but promotes exhaustion, whereas 4-1BB (CD137) sustains persistence. Dual-signaling CARs incorporating both domains such as CD28-4-1BBζ constructs demonstrate superior metabolic fitness and resistance to immunosuppressive cytokines such as TGF-β [30]. Affinity-tuned dual-targeting CARs also widen therapeutic windows; lower-affinity CD22-CARs paired with CD19-CARs selectively eliminate high-antigen-expressing tumor cells, sparing normal tissues [24].

Dual targeting, however, has several complications: limitations in the design of the vectors, possibilities of tonic signaling and challenges in production [30]. Previously, next-generation prototypes included immune-modulatory factors (e.g., PD-1-blocking scFvs or logic-gated systems to respond dynamically to the tumor environment) [31]. History highlights how the field is moving toward a combination of precision against the target of empirical antigen targeting to a synthetic biology-powered precision that aims to reveal CAR-T-cell efficacy in nonhematologic malignancies.

### Comparative analysis with other therapeutic modalities

Dual-targeting CAR-T-cell therapy represents a significant leap forward in breast cancer treatment, particularly for aggressive subtypes such as TNBC. Compared with traditional modalities like chemotherapy, endocrine therapy, and monoclonal antibody-based immunotherapy, dual-targeting CAR-T-cell approaches provide enhanced specificity, reduced antigen escape, and more durable immune responses (see Table 1). Studies demonstrate that this strategy can effectively target heterogeneous tumors that would otherwise evade single-antigen CAR-T cells, thereby overcoming one of the central challenges of solid tumor immunotherapy [32].

In TNBC models, bispecific or dual-target CAR-T designs, such as those targeting B7-H3/CSPG4 or mesothelin/NKG2D ligands, have demonstrated potent cytotoxicity and superior tumor regression compared with single-target CAR-T approaches. These dual constructs achieved broader antigen coverage and significantly reduced tumor burden in preclinical models without evident systemic toxicity [33, 34]. Furthermore, targeting combinations such as IL-13Rα2 and EphA2 have been proposed to overcome tumor resistance and improve clinical response rates, showing particular promise in HER2-enriched and TNBC subtypes [35]. Compared to conventional chemotherapy, which often leads to systemic toxicity and resistance, or endocrine therapy, which is ineffective in hormone receptor-negative cancers, dual-targeting CAR-T cells exhibit precision cytotoxicity, adaptability to tumor heterogeneity, and potential for long-term remission.

Beyond improved tumor eradication, these therapies also modify the immunosuppressive tumor microenvironment, enhancing T-cell persistence and cytokine secretion. However, challenges remain in optimizing CAR-T cell trafficking, mitigating cytokine release syndrome, and ensuring cost-effective scalability [36, 37]. When compared with immune checkpoint inhibitors, dual CAR-T therapies have demonstrated higher tumor-specific killing but currently lack the clinical maturity and accessibility of checkpoint-based regimens.

**Table 1:** Comparative analysis of Dual-Targeting CAR-T therapy versus other modalities in breast cancer

Comparison aspect	Dual-targeting CAR-T (DT CAR-T)	Comparator therapy	Key differences & outcomes	Supporting references
1. DT CAR-T vs. single-target CAR-T (ST CAR-T)				
Antigen targeting	Simultaneously targets two or more antigens (e.g., HER2 + MUC1, MSLN + NKG2DL) to overcome antigen heterogeneity and immune escape.	ST CAR-T targets a single antigen (e.g., HER2, MUC1) making it susceptible to tumor antigen loss or mutation.	DT CAR-T demonstrates enhanced cytotoxicity, reduced relapse risk, and improved tumor recognition in heterogeneous TNBC models.	[38, 39]
Efficacy in solid tumors	Improved tumor infiltration and persistence due to multi-antigen recognition and cytokine support.	Limited success in solid tumors; poor infiltration and short persistence due to suppressive TME.	DT CAR-T achieves higher survival and tumor regression in TNBC models.	[40]
Resistance & relapse	Reduces tumor antigen escape via dual engagement, minimizing recurrence.	Tumor escape common through antigen loss.	DT CAR-T reduces immune evasion compared to ST CAR-T.	[39]
Safety profile	Enhanced specificity leads to lower off-tumor toxicity.	Higher risk of cytokine release syndrome (CRS) and off-target effects.	DT CAR-T shows reduced systemic toxicity in preclinical models.	[40]
2. DT CAR-T vs. monoclonal antibodies (mAbs) and ADCs				
Mechanism of action	Employs antigen-specific cytotoxic T-cell activation and immune memory formation.	mAbs (trastuzumab, pertuzumab) rely on ADCC; ADCs (T-DM1, T-DXd) deliver cytotoxic payloads.	DT CAR-T acts as a “living drug” sustaining immune pressure, unlike transient antibody activity.	[41]
Resistance mechanisms	Dual antigen targeting bypasses HER2 downregulation and heterogeneity.	mAbs/ADCs develop resistance via antigen loss, altered signaling, or impaired uptake.	DT CAR-T resists escape mechanisms inherent to antibody-based therapies.	[42]
Target discovery potential	In silico modeling supports identification of safe dual targets for CAR-T or ADCs.	Current mAb/ADC designs are limited by antigen selectivity and off-target toxicity.	DT CAR-T offers broader combinatorial targeting adaptability.	[42]
Therapeutic durability	Long-term persistence of effector T-cells provides ongoing tumor surveillance.	Antibody effects wane post-clearance; require repeated dosing.	DT CAR-T has durable responses with single administration potential.	[41]
3. DT CAR-T vs. immune checkpoint inhibitors (ICIs)				
Mechanism of immune activation	DT CAR-T provides active tumor-targeted cytotoxicity and local immune modulation.	ICIs (e.g., pembrolizumab, atezolizumab) passively relieve immune suppression by blocking PD-1/PD-L1 or CTLA-4.	DT CAR-T can induce immune reprogramming independent of PD-L1 expression.	[43]
Tumor microenvironment resistance	Armored DT CAR-Ts secrete anti-PD-1 scFv or cytokines locally, overcoming TME suppression.	ICIs have limited effect in immune-cold tumors with low TIL or PD-L1 expression.	DT CAR-Ts show superior TIL infiltration and survival in TNBC models.	[44]
Combination potential	Combining DT CAR-T with ICIs or bispecific PD-L1/TGF-β blockers enhances efficacy.	ICI monotherapy benefits limited to select subtypes; resistance frequent.	DT CAR-T-ICI combinations achieve synergistic activation and better tumor control.	[45]
Durability of response	Persistent cytotoxicity via long-lived T-cells and localized checkpoint blockade.	ICI responses depend on existing immune priming; relapse frequent post-therapy.	DT CAR-T provides prolonged immune surveillance beyond ICI duration.	[46]

## Targeting antigens for dual-targeted CAR-T-cell therapy in breast cancer

Dual-target CAR-T-cell therapy is a promising strategy in the fight against breast cancer, aiming to overcome challenges such as tumor heterogeneity and antigen escape. This innovative approach involves engineering T cells to recognize and attack cancer cells by targeting two different antigens simultaneously [32].

### Ideal antigen selection criteria

**Fig. 5:**
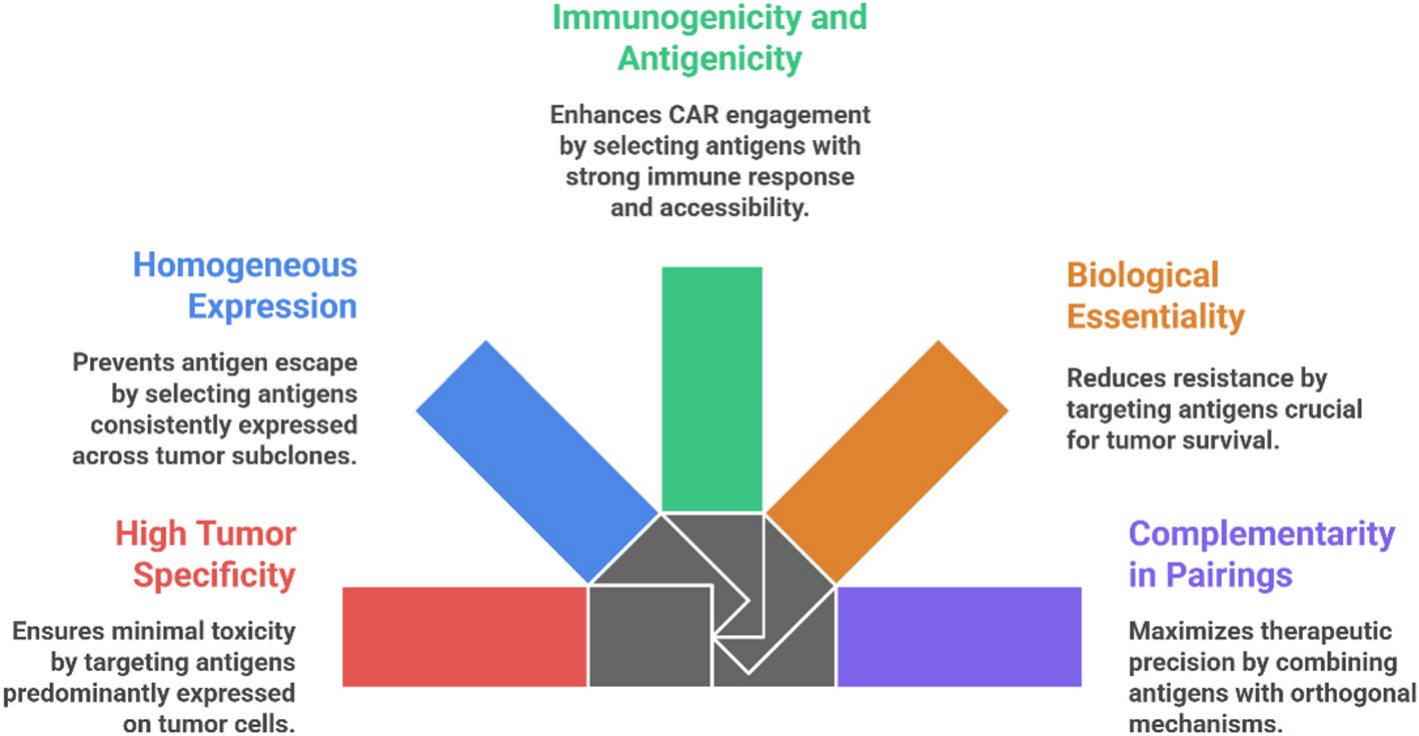
Ideal Antigen Selection Criteria for Dual-Target CAR-T-cell Therapy

The efficacy of dual-target CAR-T-cell therapy in breast cancer is critically dependent on the rational selection of target antigens, a process governed by a hierarchy of interconnected principles designed to maximize therapeutic precision while minimizing toxicity (Fig. 5). First, ideal antigens need to have high tumor specificity and overexpression; that is, the antigens are largely expressed or even restricted to tumor cells but not healthy tissues to avoid on-target off-tumor toxicity [46]. For example, although HER2 is a clear target, the limited presence of HER2 in normal tissues, such as cardiomyocytes, is still a risk of cardiac toxicity in 15–30% of breast cancer cases [47]. Targeting cancer-testis antigens or lineage-restricted antigens with minimal expression in normal somatic cells is better matched by this criterion. The other major principle involves the requirement of homogeneous expression of all tumor subclones to overcome antigen escape, which is one of the main causes of relapse in monotargeted therapies [48]. In breast cancer, targets such as MUC1 and CD70 are more consistently expressed than others that exhibit spatial heterogeneity, and dual-target strategies can mitigate this risk by pairing antigens with nonoverlapping escape mechanisms, such as oncogenes and structural proteins [7, 49].

In addition, optimal antigens must be highly immunogenic and antigenic and should be able to induce T-cell activation as well as be present in a form structurally accessible for stable binding through CARs on the cell surface, not intracellular or soluble isoforms [50]. The pivotal biological importance of the antigen for tumor survival is also crucial; hitting proteins important for proliferation or metastasis, such as the signaling node EGFR or the zinc transporter LIV-1, lowers the likelihood of the tumor downregulating the antigen in response to therapeutic pressure [51]. Finally, the success of a dual-target approach hinges on the complementarity of the chosen antigen pair, which should leverage orthogonal mechanisms to create synergy. This can be achieved through spatial complementarity, where one antigen is broadly expressed and the other is subtype specific, or by targeting antigens with different functional roles or low rates of extracellular domain shedding to ensure sustained CAR engagement. By adhering to these rigorous selection criteria, dual-target CAR-T-cell therapy can be strategically designed to overcome the key challenges associated with treating heterogeneous solid tumors such as breast cancer [52].

The introduction of antigen pairing in dual-targeting CAR-T cell therapy has emerged as a sophisticated strategy to improve the specificity and safety profile of CAR-T therapies, especially in solid tumors such as breast cancer where antigen heterogeneity and off-tumor toxicity remain major obstacles. Conventional single-antigen CAR-T therapies, such as those targeting HER2 or SSEA-4, have demonstrated potent antitumor effects but often induce severe off-tumor cytotoxicity due to the expression of these antigens in healthy tissues, as evidenced by preclinical findings where SSEA-4-directed CAR-T cells caused severe toxicity through recognition of normal progenitor cells [53]. Antigen pairing counters this limitation by employing combinatorial or “AND-gate” logic in T-cell activation, ensuring that effector functions such as cytokine secretion and cytotoxicity are triggered only when two tumor-associated antigens are simultaneously recognized [53]. This design integrates distinct signaling modules often distributing the CD3ζ and co-stimulatory (4-1BB or CD28) domains across two separate receptors thereby minimizing basal activation and reducing the likelihood of off-tumor immune responses.

In the context of breast cancer, in silico analyses have identified subtype-specific antigen pairs such as CEACAM6-GFRA1 and FGFR4-ITGB6, which display complementary expression in tumor cells with minimal overlap in normal tissue, underscoring their translational potential for precision-engineered CAR constructs [41]. Furthermore, combinatorial targeting not only enhances selectivity but also mitigates antigen escape, a common mechanism of resistance in monovalent CAR-T therapies, as demonstrated by dual-target CD19/CD22 constructs that maintained durable responses and reduced relapse risk without significant cytokine release syndrome [54]. The split-CAR and bicistronic models represent distinct engineering solutions while the split-CAR system enforces a strict AND-gate for co-expression, bicistronic CARs balance flexibility and manufacturing simplicity but may carry risks of asymmetric signaling or preferential expansion of one subset over another.

However, critical evaluation by leading experts would emphasize that the improvement in safety through antigen pairing must be balanced against practical limitations such as co-expression variability of antigen pairs, potential signal attenuation, and manufacturing complexity. Tumor heterogeneity within breast cancer subtypes could lead to therapeutic inefficacy if only one antigen is expressed in a subset of tumor cells. Moreover, the threshold tuning between activation and tolerance remains a key challenge, as low-level co-expression in normal tissues could still provoke subclinical toxicity. Thus, antigen pairing should ideally be coupled with adaptive safety mechanisms such as suicide switches, switchable receptors, or transient CAR expression to dynamically modulate T-cell activity [55].

### Common and emerging antigen targets

The effectiveness of dual-targeting CAR-T-cell therapy hinges critically on the selection of combinations of antigen pairs that can overcome tumor heterogeneity and prevent antigen escape. Widely utilized targets include HER2 (human epidermal growth factor receptor 2), which is overexpressed in 15–30% of these breast cancers and is very firmly associated with oncogenic signaling activity. HER2-targeted CAR-T cells exhibit powerful cytotoxicity, which is still limited by insufficient and responsible heterogeneous expression and adaptive resistance mechanisms in monotherapy [56, 57]. Emerging targets include MUC1 (Mucin 1), particularly its cleaved isoform MUC1*, which functions as a growth factor receptor and is overexpressed in >80% of breast carcinomas. Phase I trials of huMNC2-CAR44 T cells targeting MUC1* have shown preliminary safety and efficacy in metastatic breast cancer [58, 59]. The coexpression of EGFR (epidermal growth factor receptor) and HER2 in luminal breast cancer is particularly striking, and the combination of HER2 and EGFR blockade has a synergistic effect on tumor lysis because of the ability to overcome compensation via other pathways [60]. Another emerging target is the hepatocyte growth factor receptor c-Met, which has been implicated in metastatic progression and trastuzumab resistance, and CAR-T-cell designs targeting c-Met + breast cancer models have demonstrated activity [61].

Novel targets have specific and unique strengths: Trop2 (trophoblast cell-surface antigen 2) is widely overexpressed in TNBC and allows costimulatory CAR designs to drive up TCR-mediated cytoxics in combination with tumor antigen receptors [62]. The tight junction protein claudin 18.2, which is tumor restricted, is being evaluated in gastric cancer CAR-T cells but has preclinical applicability in claudin-low breast tumors [63]. Receptor tyrosine kinase-like orphan receptor 1 (ROR1) is an oncofetal antigen that is primarily absent from most adult cell types but constitutively expressed in breast cancer stem cells. Preclinical ROR1-CAR-T cells induce long-term remission in xenograft models by targeting tumor-initiating cells [64, 65]. The B7-H3 (CD276) immune checkpoint is a pancancer target that runs high on both the vasculature of breast tumors and cancer-associated fibroblasts. Compared with conventional CAR-T cells, B7-H3-directed CAR-T cells exhibit enhanced proliferative capacity and penetrate immunosuppressive microenvironments more effectively [14].

Antigen pair combinations under investigation leverage synergistic biological roles to mitigate escape. Notable pairs include HER2/MUC1 to target divergent molecular subtypes; EGFR/c-Met to block compensatory growth pathways; B7-H3/ROR1 to simultaneously attack stroma-protected and stem-like populations; and Trop2/Claudin 18.2 for comprehensive coverage of metastatic lesions. Compared with monovalent approaches, dual-targeting strategies employing bispecific CARs or tandem CAR architectures significantly reduce antigen escape rates (*p* < 0.01) in PDX models (see Table 2) [66, 67]. This combinatorial logic pairing established targets with emerging antigens forms the foundation for next-generation precision CAR-T-cell therapies capable of addressing the spatial and temporal heterogeneity of breast cancer.

**Table 2:** Key characteristics of common and emerging antigen targets in cancer therapy

Antigen target	Associated cancers	Therapeutic approaches	Status/key findings	Refs.
HER2 (human epidermal growth factor receptor 2)	Breast Cancer, Gastric Cancer, Gastroesophageal Junction Adenocarcinoma, Lung Cancer, Colorectal Cancer, Bladder Cancer.	Monoclonal Antibodies (e.g., Trastuzumab, Pertuzumab, Margetuximab), Antibody‒Drug Conjugates (ADCs) (e.g., T-DM1, Trastuzumab Deruxtecan), Tyrosine Kinase Inhibitors (TKIs) (e.g., Lapatinib, Neratinib, Tucatinib).	HER2-targeted therapies are well-established for HER2-positive breast and gastric cancers, significantly improving patient outcomes. The development of ADCs has transformed treatment for HER2-positive cancers, and the concept of “HER2-low” is expanding the patient population who may benefit.	[68–70]
MUC1 (Mucin 1)	Breast Cancer, Pancreatic Cancer, Ovarian Cancer, Lung Cancer, Colon Cancer.	Monoclonal Antibodies, Antibody‒Drug Conjugates (ADCs), CAR-T-Cell Therapy, Cancer Vaccines, Aptamers.	MUC1 is overexpressed and abnormally glycosylated in many cancers, making it a promising target. Various therapeutic strategies are in preclinical and clinical development, with some showing potential in early phase trials. The MUC1-C subunit is a direct target for inhibitors.	[71, 72]
EGFR (epidermal growth factor receptor)	Non-Small Cell Lung Cancer (NSCLC), Colorectal Cancer, Head and Neck Cancer, Glioblastoma, Pancreatic Cancer.	Monoclonal Antibodies (e.g., Cetuximab, Panitumumab), Tyrosine Kinase Inhibitors (TKIs) (e.g., Gefitinib, Erlotinib, Osimertinib), Peptide Vaccines.	EGFR-targeted therapies are a cornerstone of treatment for NSCLC with specific EGFR mutations. However, acquired resistance is a major challenge, leading to the development of next-generation inhibitors and combination strategies.	[73]
c-Met (hepatocyte growth factor receptor)	Non-Small Cell Lung Cancer (NSCLC), Gastric Cancer, Kidney Cancer, Ovarian Cancer, Pancreatic Cancer, Head and Neck Cancer, Colon Cancer.	Monoclonal Antibodies (e.g., Rilotumumab, Onartuzumab), Antibody‒Drug Conjugates (ADCs), Tyrosine Kinase Inhibitors (TKIs) (e.g., Crizotinib, Capmatinib, Tepotinib).	c-Met is often overexpressed in various cancers and its signaling pathway is implicated in tumor growth and metastasis. Targeting c-Met is a promising strategy, especially in cancers with MET gene amplification or mutations. Combination therapies are being explored to overcome resistance.	[74–77]
Trop2 (trophoblast cell-surface antigen 2)	TNBC, Hormone Receptor-Positive Breast Cancer, Urothelial Cancer, Lung Cancer, Prostate Cancer, Pancreatic Cancer.	Antibody‒Drug Conjugates (ADCs) (e.g., Sacituzumab Govitecan), Monoclonal Antibodies, Multispecific Agents, Cancer Vaccines, Small Molecule Inhibitors.	Trop2 is highly expressed in a variety of solid tumors with limited expression in normal tissues. The ADC Sacituzumab govitecan is FDA-approved and has shown significant survival benefits in metastatic TNBC and urothelial cancer. Numerous other Trop2-targeted therapies are in development.	[78, 79]
Claudin 18.2	Gastric Cancer, Gastroesophageal Junction Adenocarcinoma, Pancreatic Cancer, Esophageal Cancer.	Monoclonal Antibodies (e.g., Zolbetuximab), CAR-T-Cell Therapy, Antibody‒Drug Conjugates (ADCs).	Claudin 18.2 is a highly specific target as its expression is largely restricted to the stomach lining and becomes accessible in cancer cells. Zolbetuximab, a monoclonal antibody, has shown positive results in late-stage clinical trials for gastric and GEJ cancers when combined with chemotherapy.	[80, 81]
ROR1 (receptor tyrosine kinase-like orphan receptor 1)	Chronic Lymphocytic Leukemia (CLL), Mantle Cell Lymphoma, Lung Cancer, Breast Cancer, Ovarian Cancer, Pancreatic Cancer.	Monoclonal Antibodies, Antibody‒Drug Conjugates (ADCs), CAR-T-Cell Therapy, Small Molecule Inhibitors.	ROR1 is an oncofetal protein, meaning it is expressed during embryonic development and re-expressed in various cancers, with minimal expression in normal adult tissues. This makes it an attractive target for therapies with potentially fewer side effects. Several ROR1-targeted agents are in preclinical and clinical development	[82]
B7-H3 (CD276)	Prostate Cancer, Neuroblastoma, Glioblastoma, Ovarian Cancer, Colorectal Cancer, Lung Cancer, Breast Cancer.	Monoclonal Antibodies, Antibody‒Drug Conjugates (ADCs), CAR-T-Cell Therapy, Radioimmunoconjugates, Bispecific Antibodies.	B7-H3 is an immune checkpoint molecule that is overexpressed in a wide range of cancers and is associated with poor prognosis. Its limited expression in normal tissues makes it a promising target for various therapeutic approaches, which have shown anti-tumor activity in preclinical and early clinical studies.	[83, 84]
Antigen pair combinations	Various Solid Tumors (e.g., Non-Small Cell Lung Cancer, Hepatocellular Carcinoma).	Bispecific Antibodies (bsAbs), Dual CAR-T-Cell Therapy.	Targeting two antigens simultaneously can increase tumor selectivity and overcome antigen escape, a common mechanism of resistance. Examples include targeting EGFR and c-Met in NSCLC or GPC3 and MUC13 in hepatocellular carcinoma. This approach aims to create a larger therapeutic window by requiring the presence of both antigens for a therapeutic effect.	[85, 86]

## Dual-targeting CAR-T-strategies: design principles and engineering approaches

CAR-T-cell therapy has transformed how cancer can be treated, especially for hematologic malignancies. Nevertheless, limitations to single-antigen-targeting CAR-T-cell therapies are common due to issues such as tumor antigen heterogeneity and antigen loss, which can lead to relapse in patients and treatment failure [87]. To overcome these challenges, an approach involving the use of double-targeting CAR-T cells, in which T cells engineered to kill cancers expressing either of two varieties of antigens, has been developed. This approach helps avoid the danger of immune escape and increases the durability of therapies [88]. Recent innovations include bicistronic CAR constructs, tandem scFv designs, and IF/THEN-gated circuits that conditionally activate secondary CAR expression in the tumor microenvironment to increase specificity and reduce off-tumor toxicity [89]. Engineering solutions such as transposon-mediated delivery and dual-vector systems have also been developed to increase the genetic payload capacity and functional complexity of CAR-T-cell products. The combination of these advances represents an important step toward the development of more versatile CAR-T-cell therapies of the next generation that would be effective enough to respond to various and changing tumor environments [90].

Recent studies have identified mesothelin (MSLN) as a promising CAR target in TNBC due to its high tumor-specific expression and association with poor prognosis. However, single-antigen MSLN-CAR-T therapies face challenges such as antigen heterogeneity and immune suppression. To address this, allogeneic IL-15–enhanced MSLN-CAR-NKT (Allo15MCAR-NKT) cells have been developed, combining CAR-mediated cytotoxicity with innate NKT receptor activity. These cells demonstrated potent dual target killing of MSLN⁺ tumor cells and CD1d⁺ immunosuppressive macrophages, achieving strong antitumor effects and low toxicity in preclinical TNBC models. This approach highlights how multi-receptor and dual-target CAR designs can overcome the antigen escape and microenvironmental resistance limiting earlier therapies [91].

### Design principles of dual-targeting CAR-T cells

CAR-T-cell therapy with dual-targeting is a new technology aimed at enhancing the safety and efficacy of cancer immunotherapy, particularly for solid tumors such as breast cancer, and overcoming the limitations of immunotherapy, including heterogeneity and escape of antigens, via two-signal antigen specificity. Dual-targeting CAR-T cells exploit the concept of traditional CAR-T-cell therapy by modifying or engineering CAR-T cell-cells to recognize two individual tumor-associated antigens in either a sequential or simultaneous manner [92, 93]. Several key design principles underpin the development of these advanced CAR-T-cell constructs.

#### Selection of target antigens

The selection of appropriate target antigens plays an important role in the efficacy and safety of dual-targeting CAR-T-cell therapy [94]. Its ideal targets are highly expressed on tumor cells and are expressed at low levels on normal tissues to decrease off-target effects [95]. For example, scientists have investigated how to produce dual-targeting CAR-T cells against intracellular alpha-fetoprotein (AFP) and cell surface glypican-3 (GPC3) in liver cancer. By taking advantage of the excellent specificity of AFP as a biomarker of liver cancer, this strategy could attack GPC3 to better identify tumor cells [89]. In acute myeloid leukemia (AML), a gated approach to cotarget ADGRE2 and CLEC12A has been developed to selectively kill AML cells with minimal impact on normal hematopoietic stem/progenitor cells. This dual targeting of CAR-T cells by ADGRE2 and CLEC12A improves AML cell specificity and decreases the likelihood that normal cells will experience off-target effects [94].

#### Bispecific CAR receptor architecture

Bispecific CAR receptors bind two domains within a single CAR molecule. This design allows CAR-T cells to bind to two different antigens on the tumor cell surface simultaneously. Various configurations exist, including tandem CARs, where two single-chain variable fragments (scFvs) targeting different antigens are linked in tandem. Tandem CARs can also target different epitopes of the same antigen. This approach enhances the avidity of CAR-T cells for tumor cells, promoting stronger activation and cytotoxicity [96].

Recent advances in chimeric antigen receptor (CAR) design have demonstrated that dual-targeting strategies can be implemented either through co-expression of two distinct CARs containing separate single-chain variable fragments (scFvs) or via tandem CAR configurations in which two scFvs are linked within a single receptor construct. In bicistronic or dual CAR systems, each CAR is expressed independently on the T-cell surface, allowing for flexible and independent activation through each antigen-binding domain. However, this design can result in variable expression levels, potential signaling interference, and increased tonic signaling, which may influence persistence and safety. In contrast, tandem CARs (TanCARs), which integrate two scFvs within one continuous receptor, promote simultaneous recognition of two antigens through a single signaling complex, offering synchronized activation and enhanced tumor lysis, particularly in heterogeneous tumors. A comparative summary of structural, functional, and safety differences between dual and tandem CAR configurations is presented in Table 3. Studies comparing these architectures have shown that tandem CARs targeting CD19 and CD20 or CD22 elicit stronger and more consistent cytotoxicity, reduce cytokine release, and mitigate antigen escape compared to co-expressed dual CAR systems [97–99]. Furthermore, tandem configurations have demonstrated superior control of antigen-heterogeneous or antigen-loss tumors, as observed in glioblastoma and ovarian cancer models, without increasing off-target toxicity [100]. Despite these advantages, tandem CARs may present challenges related to receptor folding and optimal linker design, which can affect surface expression and signaling balance [101]. Overall, evidence suggests that tandem CAR configurations may offer improved efficacy and reduced antigen escape, with a comparable or potentially safer cytokine profile relative to separate CAR constructs.

**Table 3:** Comparison between dual CAR (Separate scFvs) and tandem CAR (Linked scFvs) constructs

Feature	Dual CAR (two separate scFvs)	Tandem CAR (two scFvs in one receptor)	Ref.
Structural design	Two distinct CAR molecules co-expressed (bicistronic vector or dual transduction)	Single CAR molecule containing two scFvs connected by a linker	[97]
Antigen recognition	Independent recognition and activation via each CAR	Simultaneous recognition through one signaling unit	[98]
Functional efficacy	May show variable cytotoxicity and signaling strength	Typically stronger and more synchronized anti-tumor response	[99]
Resistance to antigen escape	Moderate; relies on co-expression efficiency	High; dual engagement reduces escape via single antigen loss	[100]
Cytokine release/safety	Potentially higher due to dual activation of independent CARs	More regulated activation, potentially lower cytokine toxicity	[101]
Manufacturing complexity	Higher; requires dual transduction or bicistronic expression system	Lower; single construct simplifies transduction and quality control	[97]
Potential limitations	Risk of uneven CAR expression or competition between receptors	Folding or linker design can affect receptor stability	[101]

#### Coexpressed CAR systems

In coexpressed CAR systems, CARs operate independently but within the same CAR-T cells but on a single cell. This can be accomplished through either a bicistronic construct that encodes both CARs on the same mRNA transcript or cotransduction of T cells via two different vectors, each carrying a distinct CAR. When CARs bind with the corresponding antigens in tumor cells, T-cell activity is completely stimulated when both CARs are bound [102].

#### Logic-gated circuits

The logic-gating approach to CAR-T-cell therapy provides a more sophisticated solution to promote the specificity and safety of CAR-T cells: the recognition of multiple antigens is required to activate logic-gated CAR-T cells. These designs include sharper Boolean logic gates, including AND, OR, and not gates, to modulate the activities of CAR-T cells on the basis of the presence or absence of specific antigens [103]. CAR-T cells with AND gates specifically state that T cells are activated only in the presence of both antigens, decreasing the chance of off-target toxicity in healthy tissues expressing either of the targeted antigens. With this OR gate construct, CAR-T-cell activation occurs whenever any of the target antigens are expressed, improving the specificity of the target tumor and mitigating antigen escape [93]. A NOT gate design can inhibit CAR-T-cell activity when a specific antigen associated with healthy tissue is encountered, further improving safety. For example, a study developed an AND logic-gated CAR-T-cell therapy for DIPG (diffuse intrinsic pontine glioma), a deadly pediatric brain tumor, to improve tumor targeting [104].

### Manufacturing and delivery challenges

There are numerous challenges with manufacturing and delivering dual-targeting CAR-T-cell therapy to treat breast cancer, particularly TNBC, which limit its clinical application and broad accessibility. Complexities in cell engineering include achieving balanced expression of both CAR constructs to optimize target recognition and minimize T-cell exhaustion [27]. Coordinated signaling must also be maintained to ensure functional stability, while the risk of insertional mutagenesis from large-gene cassettes remains a concern, as these may interfere with native T-cell functions or trigger oncogenic activation [105]. Vector design and transduction strategies must overcome limitations in cargo capacity and efficiency. Although lentiviral vectors enable stable gene transfer, they carry risks of random integration and insertional oncogenesis, an issue similarly seen with retroviral vectors [106]. Nonviral systems such as Sleeping Beauty and PiggyBac transposons offer alternatives but remain constrained by limited efficiency and payload size, potentially affecting sustained CAR expression [105]. Likewise, CRISPR-based incorporation presents challenges related to precision, scalability, and off-target effects.

Further, differences between autologous and allogeneic CAR-T-cell products pose trade-offs. Autologous CAR-T cells are patient-specific and carry fewer immunological risks, but their production is costly, complex, and time-consuming, limiting availability for patients with rapidly progressing TNBC [107–109]. Allogeneic off-the-shelf CAR-T cells address cost and scalability but introduce risks of graft-versus-host disease (GVHD) and immune rejection, necessitating additional genetic engineering to delete T-cell receptor (TCR) and human leukocyte antigen (HLA) expression [110]. Collectively, these manufacturing and delivery bottlenecks underscore the urgent need for innovative approaches in vector design, cell engineering, and production strategies to fully realize the potential of dual-targeting CAR-T-cell therapy for breast cancer [111].

Strategies to enhance CAR-T-cell function include multitarget CAR-T cells, augmented CAR-T cells, and universal CAR-T cells. Multitargeted CAR-T cells recognize multiple antigens on tumor cells, increasing their specificity and efficiency. Armized CAR-T cells secrete cytokines such as IL-12, IL-15, and IL-18 to increase antitumor activity and CAR-T-cell survival. Universal CAR-T cells disrupt the TCR‒HLA interaction, creating “off-the-shelf” products for multiple patients. Fully humanized CAR-T cells, which use human-derived scFvs, reduce the risk of immune reactions, improving safety and efficacy [6].

The development of allogeneic CAR-T-cell therapies faces bottlenecks, including the risk of rejection and GVHD [108]. Strategies to overcome these problems include gene editing approaches such as CRISPR-Cas9 to knockout T-cell receptor (TCR) and HLA genes, preventing recognition and attack by the host immune system [110]. Investigating new targets of CAR-T-cell therapy in TNBC is essential since TNBC does not express estrogen receptor, progesterone receptor, or HER2, which are highly targeted in other subtypes of breast cancer. Phage-display peptides are used to target possible targets for CAR-T-cell therapy in the treatment of TNBC [112].

To overcome the exhaustion of T cells in solid tumors as a method to increase the persistence of CAR-T cells, studies are being directed toward targeting the phosphorylation of TCF7. CAR-T-cell therapy could also be combined with immune checkpoint inhibitors or oncolytic viruses to increase its effectiveness. Safety and efficacy are enhanced by logic gates and humanized scFvs. Additionally, normalization of the tumor vasculature and the production of higher levels of chemokines can enhance CAR-T-cell infiltration [12].

The quality and stemness of CAR-T cells are associated with better clinical responses. Placenta-derived T cells show promise as an allogeneic CAR-T-cell platform with preserved T-cell stemness and a more favorable cytokine profile. Multitargeted CAR-T cells, armored CAR-T cells, and universal CAR-T cells represent strategies to optimize CAR-T-cell design. Combination therapies may help reactivate the tumor microenvironment for CAR-T-cell therapy. These strategies include A2aR inhibitors combined with ICIs or Ovs (Table 4) [12, 113].

**Table 4:** Key challenges in the development and application of dual-targeting CAR-T-cell therapy for breast cancer, particularly TNBC, along with current strategies to overcome these barriers

Challenge	Description	Solution	References
Complex CAR design and large genetic payloads	Dual-targeting CARs (bicistronic or tandem CARs) increase genetic construct size, complicating viral vector packaging, transduction efficiency, and cost.	Nonviral delivery systems such as bioengineered mammalian transposons (e.g., bMLT transposon) enable the integration of large DNA cargo (>100 kb), overcoming size limitations and reducing oncogenic risks from viral vectors.	[88]
Antigen heterogeneity and escape	Breast tumors exhibit variable expression of target antigens, increasing the risk of tumor escape if one antigen is lost.	Dual-targeting CARs (e.g., targeting HER2 + MUC1) can reduce antigen escape. Furthermore, using logic-gated CAR designs (AND/OR gates) enhances specificity while maintaining activity against heterogeneous tumors.	[7, 114]
Limited tumor infiltration and delivery barriers	Solid tumors like breast cancer restrict CAR-T-cell trafficking due to dense stroma and immunosuppressive barriers.	Regional delivery strategies, including intratumoral or intraventricular administration, improve CAR-T accumulation in tumors. Chemokine receptor engineering can also promote T-cell homing.	[115, 116]
Immunosuppressive tumor microenvironment (TME)	Breast cancer’s TME inhibits CAR-T-cell function via factors like TGF-β, PD-L1, and hypoxia.	Armored CAR-T cells that secrete pro-inflammatory cytokines (e.g., IL-12) or block inhibitory signals (e.g., PD-1 dominant-negative receptors) can sustain activity in hostile TMEs.	[117]
On-target, off-tumor toxicity	Breast cancer antigens are sometimes present on healthy tissues, raising the risk of unintended cell damage.	Logic-gated CARs (e.g., SynNotch systems) activate CAR expression only in the presence of two tumor antigens, reducing damage to normal tissues.	[114, 118]
Manufacturing scalability	Current CAR-T production is autologous, personalized, and labor-intensive, limiting scalability.	Allogeneic (off-the-shelf) CAR-T products, combined with gene-editing to prevent graft-versus-host disease, can improve scalability and reduce costs.	[115]
Safety concerns (CRS and neurotoxicity)	Dual-targeted CARs may increase the risk of cytokine release syndrome (CRS) and neurotoxicity due to higher activation levels.	Inducible CAR systems (e.g., drug-switchable CARs) allow controlled activation, improving safety profiles. Incorporating suicide genes provides an emergency shutdown option.	[119]

## Tumor heterogeneity and the microenvironment: dual-targeting and CAR-T engineering strategies as complementary solutions

### Overview of tumor heterogeneity and microenvironmental barriers in breast cancer

The clinical success of CAR-T-cell therapy in breast cancer remains limited by two interconnected resistance mechanisms: tumor heterogeneity and the immunosuppressive tumor microenvironment (TME). Heterogeneity arises at multiple biological levels interpatient, intratumoral, spatial, and temporal leading to variable antigen expression and promoting immune escape following single-target therapies [38]. This diversity drives differential responses among breast cancer subtypes such as luminal, HER2-enriched, and triple-negative disease, where antigen loss variants often emerge under therapeutic pressure. Dual-target CAR-T-cell strategies address this challenge by broadening antigenic coverage, reducing the likelihood of clonal escape, and enhancing overall cytotoxic persistence [39].

Beyond intrinsic heterogeneity, the TME imposes formidable physical, metabolic, and immunologic constraints on CAR-T-cell trafficking, activation, and survival. Breast cancer microenvironments are enriched with inhibitory cytokines (TGF-β, IL-10), myeloid-derived suppressor cells (MDSCs), and cancer-associated fibroblasts (CAFs), which collectively limit immune infiltration and promote exhaustion [117]. Engineering “armored” CAR-T cells capable of secreting cytokines such as IL-12 or expressing dominant-negative TGF-β receptors has shown preclinical success in reversing this suppression and restoring effector function [120].

Therefore, combining dual-target CAR-T-cell strategies to counter antigenic diversity with engineered CAR constructs to overcome TME-induced suppression represents a complementary, layered therapeutic framework. This integrative approach has the potential to achieve more durable, widespread tumor eradication in breast cancer, addressing both tumor-intrinsic and extrinsic barriers [121].

- Molecular Subtype Heterogeneity

The discovery of the molecular classification of breast cancer into 4 distinct subtypes, Luminal A, Luminal B, HER2-enriched, and basal-like (triple-negative), is an early structure with interpatient heterogeneity. This subtype-specific classification has been refined considerably since the 2000 s, as high-throughput studies have revealed that each of these subtypes has different biological behaviors, responses to therapy and clinical outcomes. More recent studies have further elucidated these stratifications and were able to further classify TNBC into four tumor-specific subtypes, namely, BL1, BL2, M, and LAR, all of which have distinct ontologies and differential effects from those of standard-of-care chemotherapy [122].

These molecular subtypes have highly significant prognostic importance. Tumors that behave like luminal A tumors have the best results, with a local control of 99.1% and an overall survival of 95.1% in five years. Triple-negative tumors, on the other hand, exhibit the worst outcomes (89.6% rate of local control and 78.5% overall survival) [123]. Such spectacular clinical heterogeneity is indicative of the biological complexity of this condition and justification of subtype-specific treatment methods.

- Advanced single-cell genomic insights

Recently, new single-cell genomics techniques have revealed a greater degree of information concerning the heterogeneity of breast cancer than ever before [124]. The results of single-cell RNA sequencing studies revealed great intratumoral heterogeneity of breast cancer cells, involving canonical aspects such as the intrinsic subtype, expression of hormone receptors, drug targets, and drug resistance patterns. These technologies have already shown that even in single tumors, there is considerable diversity in cell types, which can affect the response to treatment and disease course [125].

Notably, single-cell studies have disrupted the ideologies of breast cancer stem cells (BCSCs), questioning the presence of a single population of BCSCs, but tumors harbor lineage-specific tumor-propagating cells (TPCs) [126]. The implications of these findings are extremely important with respect to targeted therapy because they imply that tumor hierarchies must be selected according to the cellular composition.

- Spatial and temporal heterogeneity

Spatial transcriptomic strategies and multiregional sampling studies have revealed the spatial structure of breast cancer heterogeneity. These studies have shown that breast cancers possess multiple distinct cellular neighborhoods of various cell types with different molecular features, such as distinct clusters of tumor cells, stromal cells, and immune cells. Sequencing of triple-negative breast cancers revealed by multiregional studies that genomic intratumor heterogeneity in the copy number status of a variety of driver oncogenes is correlated with resistance to treatments and progression [127].

Temporal heterogeneity adds another layer of complexity, as biomarker expression can change significantly between primary and metastatic lesions. Studies have reported rates of 16–33.6% for estrogen receptor (ER), 32–40% for progesterone receptor (PR), and 10–15.7% for HER2 between primary and metastatic tumors. This temporal evolution has profound clinical implications, as patients with discordant ER statuses between primary and metastatic tumors have a 48% increased risk of death [122].

#### Antigen escape and clonal evolution

- Mechanisms of immune evasion

The mechanisms of antigen escape in breast cancer are multifaceted and include complex interactions between cancer cells and immunity. The downregulation of HLA class I is a vital immune evasion mechanism, and studies have revealed that HLA-I loss of heterozygosity (LOH) is significantly related to poor outcomes in triple-negative breast cancer patients [128, 129]. HLA-I topography differs greatly among subtypes of breast cancer, whereby high HLA-I expression and gain or loss of HLA-I are observed more often in triple-negative tumors [128].

Recent studies have revealed that immune-edited clones can avoid detection to suppress the antigen presentation machinery (including HLA class I molecules) [129]. This is especially applicable in the case of immunotherapy, where one can then expect the development of resistant strains after initial responses, which might have lost target antigens or rather fit into other defenses to escape immune attack.

- Clonal evolution dynamics

The dynamics of clonal evolution in breast cancer have been extensively characterized through advanced sequencing technologies. Single-cell genome sequencing studies have demonstrated that the evolution of breast cancer adheres to a punctuated evolution model; copy number alterations, which are early events in tumor development, induce rapid bursts that are then gradually accumulated by point mutations, leading to the development of large clonal diversities [130].

This evolutionary model has important implications for understanding therapeutic resistance. The data show that no two single tumor cells are genetically identical, with extensive subclonal and de novo mutations contributing to clonal diversity. Compared with ER-positive tumors, triple-negative breast cancers exhibit particularly high mutational rates (13.3X higher than normal), suggesting increased clonal diversity and evolutionary potential [130].

- Advanced Tracking Technologies

Recent clonal tracking technologies have solved the technical issues involved in tracking rare clones, which can constitute as little as 0.1% of the tumor cell population. Multipatient-specific (MPT) single-cell DNA sequencing technologies have been generated to characterize information-rich mutational sites in many cells and thus permit the inference of mutational lineages and early driver events. Technologies have demonstrated that the vast majority of breast tumors are composed of 1/4 large clonal subpopulations with related progression lineages, suggesting the existence of single progenitor cells [131].

Circulating tumor DNA (ctDNA) analysis has emerged as a powerful tool for tracking clonal evolution in real time. Recent studies have introduced the concept of the tumor clonal evolution rate (TER) as a novel biomarker that reflects the speed of clonal evolution and is correlated with treatment efficacy and prognosis. Patients with lower TER values have better progression-free survival and overall survival, providing new evidence for the clinical utility of evolutionary dynamics in predicting treatment outcomes [132].

### Overcoming heterogeneity with dual-target CAR-T cells

Dual-target CAR-T-cell approaches can greatly increase the size of the immunologic net by allowing each genetically engineered T-cell or a succession of CAR-T-cell combinations to attack tumor cells expressing either of two antigens, reducing the possibility that escape variants downregulate one of these molecules. Initial trials with tandem or bicistronic CD19/CD22, CD19/CD20 and BCMA/GPRC5D constructs returned high overall and complete response rates and few relapses caused by antigen loss, which demonstrates their strength in overcoming inter- and intratumoral heterogeneity [133, 134]. Mechanistically, costimulatory, dual engagement reinforces signaling and supports the generation of more stable, bivalent immune synapses, which could translate into increased depth of cytotoxicity and extended remission [32, 135]. Nonetheless, the characteristics that extend coverage can also engender some novel risks: in addition to inflating the size of the vectors themselves, adding two scFvs reduces transduction efficiency and introduces the risk of creating mixed subpopulations of CAR-T cells expressing just one receptor, potentially recreating single-antigen vulnerability, which the approach seeks to avoid. Dual signaling can also heighten tonic activation, accelerating T-cell exhaustion and, in some cohorts, intensifying cytokine release or neurotoxicity safety signals that demand stricter target selection and rational tuning of costimulatory domains. From a translational perspective, manufacturing complexity and cost rise sharply, yet long-term follow-up remains sparse, leaving unanswered whether dual-target designs can sustain functional persistence once selective pressure on each antigen diminishes. In summary, dual-target CAR-T cells provide a powerful, conceptually elegant hedge against antigen heterogeneity, but their ultimate clinical value will hinge on overcoming engineering and safety hurdles and generating durable efficacy data that justify the added complexity [136].

### Tumor microenvironment barriers

The tumor microenvironment (TME) in breast cancer presents significant immunosuppressive and physical barriers that hinder therapeutic efficacy. Key immunosuppressive factors TGF-β, IL-10, and PD-L1 drive immune evasion but exhibit context-dependent limitations. TGF-β, produced by cancer-associated fibroblasts (CAFs) and regulatory T cells (Tregs), suppresses cytotoxic T-cell function and promotes epithelial‒mesenchymal transition (EMT) in breast cancer cells. Nevertheless, TGF-β blockade therapy is usually not effective in the clinic because of its paradoxical inhibitory effects on early carcinogenesis. Similarly, M2 macrophages and Tregs secrete IL-10, which can halt antigen presentation and the differentiation of dendritic cells [137, 138]. Similarly, IL-10 secretion by M2 macrophages and Tregs inhibits antigen presentation and dendritic cell maturation [139]. Although in preclinical models, IL-10 inhibition triggers enhanced T-cell function, there is a concern of systemic toxic autoimmune activity with systemic IL-10 inhibition, demonstrating difficulties in translation. PD-L1 on tumor cells and myeloid-derived suppressor cells (MDSCs) interacts with PD-1 on T cells, which causes exhaustion [140]. Although this axis is targeted by immune checkpoint inhibitors (ICIs), primary resistance occurs in approximately 70–80% of breast cancer patients and is caused by compensatory activation of alternative checkpoints (e.g., TIM-3 and LAG-3) [141].

Recent studies highlight that these immune checkpoints function as overlapping escape mechanisms. TIM-3 and LAG-3 are often coexpressed with PD-1 in exhausted T cells, and their blockade is required to fully restore effector function in resistant tumors [142]. Dual or sequential inhibition of PD-L1 with these alternative checkpoints is being explored to overcome resistance in breast cancer.

**Fig. 6:**
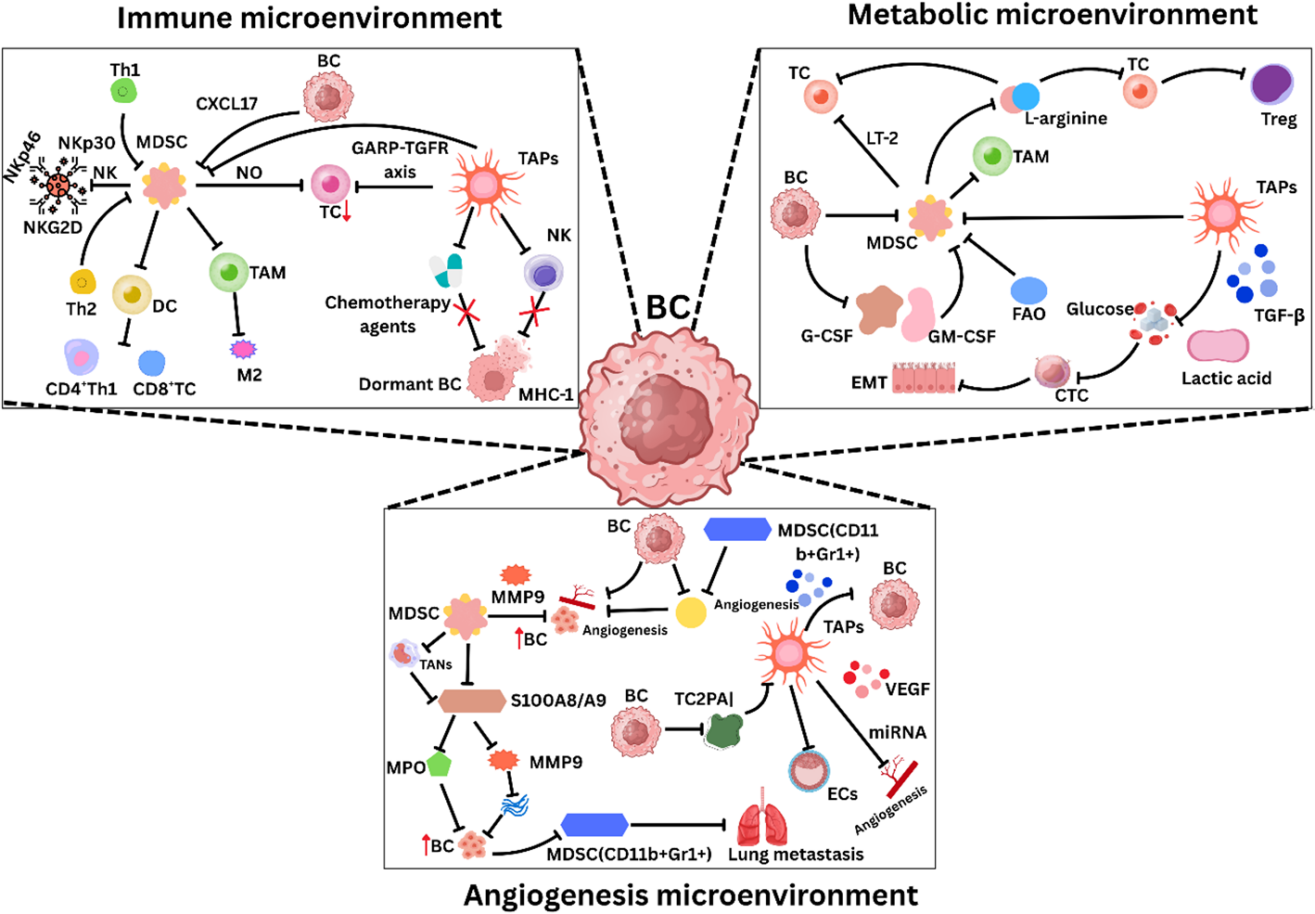
**A** Immune suppression by MDSCs and TAPs: In the breast cancer (BC) microenvironment, myeloid-derived suppressor cells (MDSCs) and tumor-associated platelets (TAPs) play critical roles in suppressing antitumor immune responses. They inhibit T-cell and natural killer (NK) cell activity, release immunosuppressive cytokines such as interleukin-10 (IL-10) and transforming growth factor-beta (TGF-β), and stimulate regulatory T-cell (Treg) expansion. These processes collectively create an immune-privileged niche that facilitates BC lung metastasis.** B** Metabolic dysregulation in BC progression: The breast cancer metabolic microenvironment, characterized by disruptions in glucose, amino acid, and lipid metabolism, significantly contributes to tumor immune evasion. These metabolic alterations reshape the tumor microenvironment, promoting immunosuppression and allowing cancer cells to escape immune surveillance.** C** Angiogenesis, dormancy, and metastatic progression: MDSCs and TAPs also drive angiogenesis and increase vascular permeability, enabling BC cells to enter a state of dormancy, survive in circulation, and colonize distant organs. This process not only increases the risk of breast cancer recurrence but also promotes tumor cell proliferation and lung metastasis

MDSCs and tumor-associated platelets (TAPs) further contribute to breast cancer immune evasion by inhibiting T-cell and NK-cell activity, secreting immunosuppressive cytokines such as IL-10 and TGF-β, and promoting regulatory T-cell expansion. In parallel, metabolic dysregulation in the breast cancer microenvironment, including alterations in glucose, lipid, and amino acid metabolism, reshapes immune responses and supports tumor progression. Additionally, MDSCs and TAPs promote angiogenesis and vascular remodeling, allowing breast cancer cells to enter dormancy, evade immune surveillance, and metastasize to distant organs, particularly the lungs (Fig. 6).

Emerging evidence suggests that targeting CXCR2, which mediates MDSC recruitment and angiogenic signaling, can reduce immune suppression and enhance checkpoint blockade efficacy [143]. Hypoxia, driven by aberrant vasculature and CAF-mediated ECM remodeling, further leads to immunosuppression. Breast cancers with hypoxic regions induce increased CAF production of TGF-β and IL-6 to increase stromal barrier formation [144, 145]. CAF-derived collagen/hyaluronan-induced ECM stiffness physically blocks T-cell infiltration and promotes prosurvival signaling pathways (e.g., HIF-1α and YAP) in cancer cells. Hypoxia also reduces drug perfusion and promotes stemness in TNBC [146]. Stromal barriers remain inadequately addressed in clinical strategies; CAF-depleting therapies often trigger reactive fibrosis, exacerbating treatment resistance [147].

Recent single-cell analyses have identified distinct CAF subsets that regulate TGF-β activity spatially and modulate CD8 + T-cell infiltration, underscoring that CAF targeting should focus on biological processes (e.g., TGF-β modulation, ECM stiffness) rather than single cell types [148]. Novel therapeutic platforms such as latent TGF-β1 inhibitors (SOF10) selectively suppress TGF-β activation in CAFs, facilitating T-cell infiltration without systemic toxicity [149]. Likewise, FAP and DDR1 inhibitors remodel the collagenous ECM and reduce CAF-derived IL-6 and IL-8 secretion, creating a more immune-permissive microenvironment [150].

Implications: These barriers necessitate multipronged targeting. Disrupting TGF-β or PD-L1 requires combination with stroma-modifying agents (e.g., hyaluronidase, CXCR2 inhibitors) to alleviate hypoxia and enhance immune cell access [151]. Future approaches must stratify patients by TME biomarkers (e.g., CAF subsets and spatial immune cell distribution) to optimize barrier-specific interventions [152]. Integration of CAF-modulating agents, such as FAP inhibitors or latent TGF-β1 blockers, may enhance immune checkpoint therapy by normalizing the stroma and improving CAR-T cell infiltration.

### Engineering CAR-T cells to combat micro-environmental resistance

Engineering CAR-T cells to overcome the immunosuppressive tumor microenvironment (TME) in breast cancer employs three primary strategies: dominant-negative receptor (DNR), cytokine-secreting “armored” CAR-T cells (e.g., IL-12/IL-18), and checkpoint inhibitor coexpression. Each approach presents unique strengths, weaknesses, and implications for clinical translation. The breast cancer TME imposes multiple suppressive mechanisms that limit CAR-T cell efficacy, including cytokine-mediated inhibition (TGF-β, IL-10), immune checkpoint signaling (PD-L1, TIM-3, LAG-3), and stromal or metabolic barriers driven by cancer-associated fibroblasts (CAFs), ECM stiffness, and hypoxia-associated HIF-1α and YAP pathways [153–156]. These factors collectively restrict T-cell infiltration, proliferation, and persistence, underscoring the need for engineering strategies that counter these TME-specific obstacles [157].

Dominant-negative receptors such as TGFβRII-DNR operate in an interception signaling behavior that blocks inhibitory molecules, including TGF-B, which is common in the TME of breast cancer [6]. Although the in vitro use of DNRs has been proven to improve CAR-T-cell proliferation and cytotoxicity, their application in clinical models of breast cancer has not yet been proven. The drawbacks are multiple immunosuppressive pathways (IL-10, PGE2) and potential unintended signaling crosstalk [12]. Armored CAR-T cells that produce IL-12 or IL-18 target TME remodeling. IL-12 production converts macrophages, eliminates immunosuppressive cells (MDSCs, TAMs) and infiltrates intrinsic immunity, enhancing the control of breast cancer in murine models [158]. Nonetheless, there are high risks of toxicity (e.g., cytokine release syndrome) with systemic IL-12 exposure, and IL-18-derived IFN-alpha can intensify T-cell exhaustion. Fibers of TRUCK (T cells redirected for universal cytokine-mediated killing) platforms endowed with inducible cytokine expression (e.g., iIL-12) provide safer precision, but their generation is complicated [159].

Checkpoint inhibition of coexpression (e.g., PD-1dn and CTLA-4 scFv) locally disables immune evasion. Breast CAR-T cells coexpressing PD-1dn show improved persistence in PD-L1(^+) environments, yet antigen heterogeneity in breast tumors permits escape via alternative checkpoints (e.g., TIM-3 and LAG-3). Additionally, continuous checkpoint blockade may drive T-cell dysfunction over time [160].

### Addressing tumor microenvironment (TME) resistance mechanisms

The breast cancer tumor microenvironment (TME) presents formidable barriers that impede CAR-T cell infiltration, persistence, and cytotoxic function. Components such as regulatory T cells (Tregs), myeloid-derived suppressor cells (MDSCs), tumor-associated fibroblasts (CAFs), and immunosuppressive cytokines like TGF-β and IL-10 create an inhibitory milieu that limits CAR-T efficacy [36]. Dual-target CAR-T (DT CAR-T) systems have been specifically designed to circumvent these microenvironmental constraints by integrating multiple mechanisms of resistance and activation within a single therapeutic construct. For instance, B7-H3/CSPG4-targeted DT CAR-T cells demonstrated robust antitumor activity in desmoplastic breast tumors by simultaneously targeting tumor and stromal components, thereby reducing ECM density and improving intratumoral infiltration [33].

Moreover, armored DT CAR-T constructs engineered to secrete IL-12, IL-15, or anti-PD-1 single-chain fragments (scFv) have shown the ability to locally modulate the TME by reprogramming suppressive myeloid cells and Tregs into proinflammatory phenotypes. This dual-action approach enhances T-cell persistence and reduces systemic toxicity compared to systemic checkpoint blockade [43, 44]. Additionally, logic-gated or SynNotch-based DT CAR-T designs enable activation only in the presence of dual tumor antigens, reducing off-tumor activation while allowing selective cytokine release and localized TME modulation [161].

In addressing metabolic challenges, DT CAR-T cells have been optimized to resist hypoxic and nutrient-deprived conditions typical of solid tumors. Constructs incorporating hypoxia-inducible promoters and enhanced mitochondrial metabolism (via PGC-1α expression) have demonstrated sustained cytotoxicity and viability under low-glucose, low-oxygen conditions [38]. Furthermore, some DT CAR-T designs co-targeting MSLN and NKG2D ligands induce the secretion of bispecific T-cell engagers (BiTEs) that recruit endogenous T cells and natural killer (NK) cells into the tumor site, amplifying immune infiltration even in immunologically “cold” TNBC models [34].

Collectively, these strategies highlight the next-generation design philosophy of DT CAR-T therapy, which not only targets tumor cells but also actively remodels the breast cancer microenvironment. By simultaneously addressing immunosuppression, stromal density, and metabolic stress, dual-target CAR-T systems demonstrate the capacity to convert resistant tumor niches into immune-permissive environments marking a critical advancement toward achieving durable and systemic tumor clearance in solid breast malignancies.

## Clinical landscape of dual-target CAR-T cells in breast cancer

The clinical landscape of dual-target CAR-T-cell therapy in breast cancer represents an evolving frontier in cancer immunotherapy, with significant growth in clinical trials and promising early efficacy signals, although challenges remain in achieving optimal therapeutic outcomes.

Before the emergence of dual-target strategies, several tumor-associated antigens had been explored as single CAR-T targets in triple-negative breast cancer (TNBC), among which Mesothelin (MSLN) has gained substantial attention. MSLN is selectively overexpressed in a majority of TNBC tumors while largely absent in normal tissues, establishing it as a safe and tumor-specific target [162]. Preclinical and translational studies have shown that MSLN-directed CAR-T cells induce potent cytotoxicity against TNBC models, with improved therapeutic responses following microenvironmental modulation and optimized antigen presentation [163]. Moreover, innovative platforms such as bispecific or BiTE-secreting MSLN-CAR-T constructs have demonstrated enhanced tumor eradication and reduced antigen escape by engaging multiple immune pathways [34]. These findings highlight the translational value of MSLN as a key single-target model that has informed the design of next-generation dual-target CAR-T therapies, which aim to overcome the heterogeneity and adaptive resistance frequently observed in TNBC.

### Clinical trial activity and enrollment

The current clinical landscape encompasses over 22 active clinical trials evaluating CAR-T-cell therapies in breast cancer, with dual-target approaches representing approximately 15–20% of the total studies. The total planned enrollment across all breast cancer CAR-T trials exceeds 1,000 patients, with major trials including NCT04650451 (HER2, *n* = 220) [164], NCT02414269 (mesothelin, *n* = 179) [165], and NCT04025216 (TnMUC1, *n* = 112). Notably, the NCT04430595 trial represents the largest dual-target study, targeting HER2/GD2/CD44v6, with 100 patients enrolled. Current dual-target clinical trials demonstrate geographic concentration primarily in the United States and China, with limited global accessibility reflecting manufacturing and regulatory complexities. The majority of trials (85%) are phase I studies, indicating the early developmental stage of this therapeutic approach [166].

### Response rates and efficacy outcomes

Clinical data reveal variable response rates across different CAR-T-cell approaches in breast cancer. HER2-targeted CAR-T-cell therapies have overall response rates ranging from 50 to 83.3%, whereas MUC1-targeted approaches have response rates ranging from 60 to 65%. Dual-target strategies, particularly CD19/CD20 CAR-T cells in hematologic malignancies, achieve response rates of 72.7%, with complete remission rates of 63.6%. For solid tumors, the overall pooled response rate for CAR-T-cell therapy is 9% (95% CI: 4–16%), with complete response rates of 2.3% and partial response rates of 9.5%. However, dual-target approaches show enhanced efficacy, with response rates reaching 62–72% in specific breast cancer subtypes. EGFR-targeted CAR-T cells demonstrate potent antitumor activity in triple-negative breast cancer, with preclinical studies showing 75% overall response rates and 40% complete response rates. MUC1 CAR-T-cell therapy shows particular promise in TNBC, with clinical trials reporting 65% overall response rates and 35% complete response rates [166–168].

### Safety profile and adverse events

Dual-target CAR-T constructs have emerged as a promising strategy to mitigate antigen escape in solid and hematologic cancers, yet their influence on treatment safety, particularly cytokine release syndrome (CRS) and neurotoxicity, remains complex. In the context of breast cancer, where the immunosuppressive tumor microenvironment and heterogeneous antigen expression pose challenges, dual-target CAR-T designs can alter cytokine dynamics and neural toxicity profiles in both beneficial and risky ways. Preclinical and clinical findings suggest that dual targeting may reduce severe CRS compared to monospecific CAR-T therapies by distributing activation signals and preventing hyperactivation of single antigen pathways. For instance, CD19/CD22 dual-target CAR-T constructs demonstrated significantly reduced grade 3–4 CRS (only one severe case among 16 patients) and no neurotoxicity, highlighting an improved safety profile linked to moderated cytokine kinetics [169]. Similarly, bicistronic CD19/CD22 CAR-T cells in pediatric leukemia showed no severe CRS or neurotoxicity, suggesting that dual targeting can maintain anti-tumor efficacy while curbing inflammatory overload [54].

Mechanistically, cytokine storm severity is largely driven by IL-6, IFN-γ, and TNF-α release from activated CAR-T and macrophage populations, which induce endothelial activation and vascular permeability, triggering CRS and neurotoxicity [170, 171]. Dual-target constructs, by distributing T-cell activation across multiple antigens, may reduce cytokine overload from over-engaged single-antigen CAR-Ts. Moreover, evidence from mechanistic reviews indicates that ICANS (immune effector cell–associated neurotoxicity syndrome) correlates with systemic cytokine surges and endothelial disruption rather than direct neuronal antigen targeting [172, 173].

In solid tumors like breast cancer, preclinical models suggest that dual-target CAR-Ts may mitigate off-tumor cytotoxicity through selective targeting of co-expressed antigens, such as HER2 and MUC1, limiting systemic cytokine release and improving safety margins [174]. However, cytokine modulation remains unpredictable due to tumor burden, co-stimulatory domain design, and host immune status. High-affinity dual-target constructs may inadvertently enhance cytokine cascades if both targets are densely expressed within the tumor, emphasizing the need for tunable signaling strength [175].

Overall, dual-target CAR-T cells appear to improve therapeutic safety by modulating activation thresholds, reducing single-antigen overactivation, and lowering the incidence of severe CRS and neurotoxicity. Yet, their performance in solid tumors like breast cancer requires careful antigen pairing and cytokine monitoring to balance efficacy and immune-mediated toxicity.

Compared with that of hematologic malignancies, the safety profile of dual-target CAR-T-cell therapy in breast cancer patients demonstrates manageable toxicity profiles with distinct patterns. Cytokine release syndrome (CRS) occurs in 8.3–63.6% of patients, with grade 3 or higher CRS rates remaining low at 0–10%. Immune effector cell-associated neurotoxicity syndrome (ICANS) rates are generally lower in solid tumors, occurring in 0–15% of patients. limiting toxicity, ICANS, or serious adverse events reported in recent phase I studies. Grade 3–4 adverse events occur in 16.7–30% of patients, with treatment discontinuation rates ranging from 0 to 10%. Off-tumor toxicity remains a significant concern, particularly for EGFR-targeted approaches where careful monitoring of normal tissue expression is essential.

### Survival outcomes and durability

**Fig. 7:**
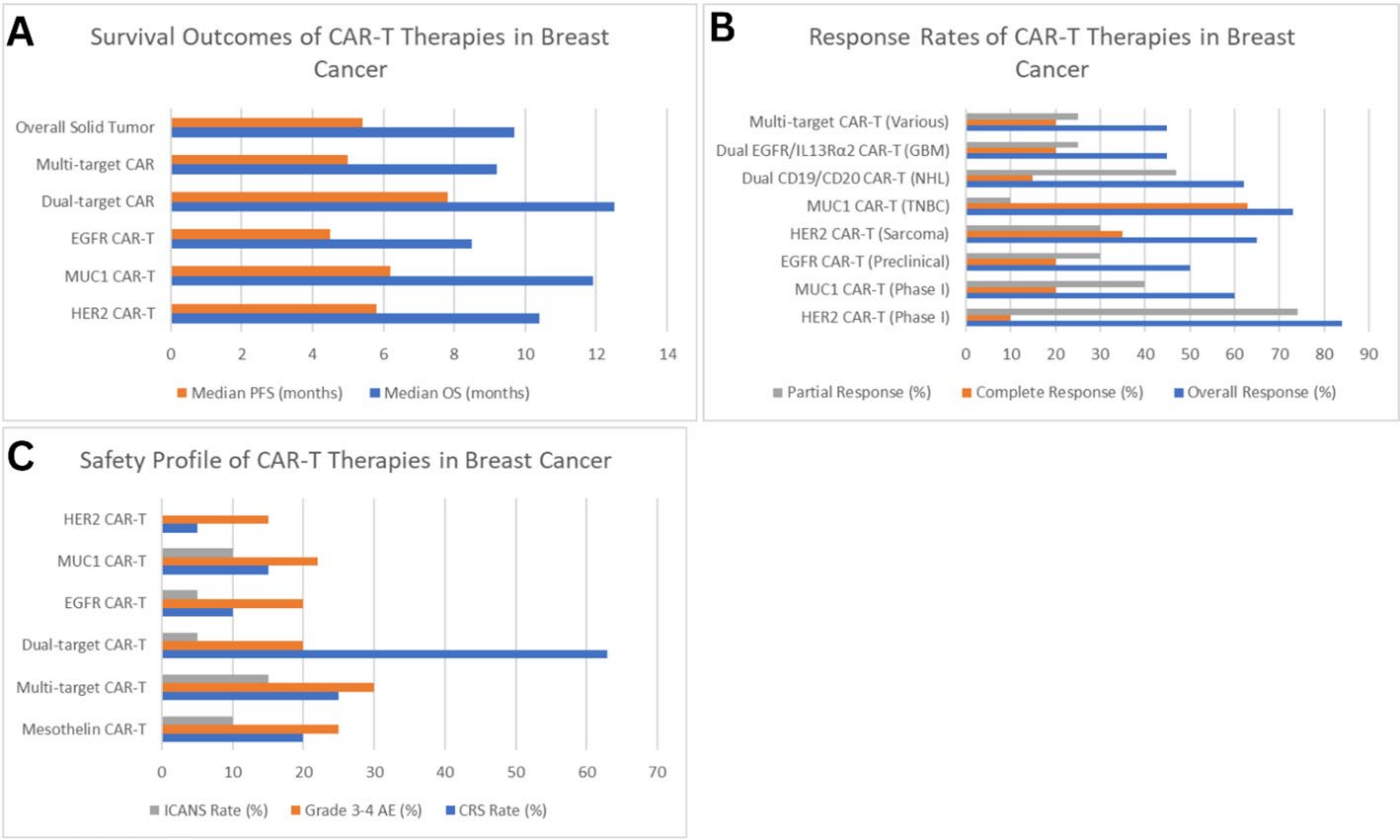
Clinical outcomes and safety profile of CAR-T-cell therapies in breast cancer.** A** Survival outcomes: Median progression-free survival (PFS) and overall survival (OS) across different CAR-T-cell lines in patients with breast cancer. Compared with other CAR-T-cell therapies, dual-target CAR-T-cell and HER2 CAR-T-cell therapies result in longer OS.** B** Response rates: overall response rate (ORR), partial response (PR), and complete response (CR) rates for various CAR-T-cell therapies targeting breast cancer antigens, including MUC1, HER2, EGFR, and dual/multitarget CARs [177]. MUC1 CAR-T cells have the highest ORR in TNBC, whereas HER2 CAR-T cells have promising early-phase results.** C** Safety profile: Incidence of immune effector cell-associated neurotoxicity syndrome (ICANS), grade 3–4 adverse events (AEs), and cytokine release syndrome (CRS) for different CAR-T-cell therapies in patients with breast cancer. Compared with single-target CAR-T-cell therapies, multitarget CAR-T-cell approaches exhibit higher toxicity rates, particularly for CRS [178]

The median overall survival (OS) of patients receiving CAR-T-cell therapy for breast cancer ranges from 8.5 to 12.5 months, with dual-target approaches resulting in superior outcomes compared with single-target strategies, as shown in Fig. 7. The median progression-free survival (PFS) ranges from 4.5 to 7.8 months, with six-month OS rates ranging from 70 to 85% and twelve-month OS rates ranging from 40 to 65%. Compared with single-target approaches, dual-target CAR-T-cell therapy has enhanced durability, with a median OS of 12.5 months and a median PFS of 7.8 months. HER2 CAR-T-cell therapy has a median OS of 10.3 months in sarcoma patients, with 50% of patients maintaining stable disease for 12 weeks to 14 months [176].

## Challenges and opportunities

Dual-targeting CAR-T-cell therapy for breast cancer confronts major hurdles including complex vector design, extended manufacturing timelines, stringent quality control, increased toxicity risk, and formidable TME barriers while offering transformative opportunities through advanced engineering, manufacturing innovations, combination regimens, personalized medicine, and regulatory strategies.

The complicated nature of the manufacturing of dual-scFv constructs substantially decreases the transduction efficiency (65–75% vs. 92–98% for single targets) and exceeds 200 labor hours per batch, initiating vein-to-vein times to 18–28 days and increasing the cost to $500,000–$700,000 per treatment. The need to achieve balanced expression of two CARs is also a concern in terms of insertional mutagenesis and makes the choice of vectors more complicated, as lentiviral, retroviral and transposon-based systems must be considered [179, 180]. Clinically, safety risks such as enhanced cytokine release syndrome and neurotoxicity due to dual CAR signaling necessitate complex dose and monitoring regimens; expanded antigen spans additionally pose a safety threat posing an on-target, off-tumor danger and necessitate in-depth normal-tissue characterization [181]. The selection of patients is still in its early stages, and there are no uniform standards of dual-antigen biomarker tests that can be used to predict responses or toxicity [182]. Within the tumor microenvironment, CAF- and MDSC-driven TGF-β/IL-10 secretion, hypoxia-induced ECM remodeling, and nutrient competition severely limit CAR-T-cell infiltration, persistence, and function, necessitating combination approaches to disrupt immunosuppressive networks and physical barriers [183].

Conversely, innovation pathways involve SynNotch and modular universal CAR platforms, which allow logic-gated or tumor-induced activation; bacteria-like CAR-T cells are engineered to secrete IL-12/15 to modify the TME; and third-generation CARs are designed to incorporate multiple signaling moieties responsible for the generation of costimulatory agents, thereby further prolonging CAR-T-cell persistence and cytotoxicity [136]. Production could be simplified and reduced in cost by automated closed-system bioreactors, point-of-care manufacturing, and AI/ML-based QC, and nonviral delivery methods (PiggyBac, mRNA) have the potential to rapidly scale production [179].

Recent innovations in CAR-T cell manufacturing have significantly improved scalability, safety, and affordability through the adoption of closed-system automated bioreactor platforms such as CliniMACS Prodigy and Lonza Cocoon, which reduce manual labor, contamination risk, and production timelines by up to 40% [184]. Nonviral gene-transfer systems like PiggyBac and Sleeping Beauty have enabled virus-free, scalable CAR-T production at only 10–20% of the cost of lentiviral methods [185], while CRISPR-Cas9-based allogeneic “off-the-shelf” CAR-T cells further lower patient-specific costs and manufacturing complexity [186]. Integrating AI-driven analytics and machine learning has improved process control by predicting optimal transduction windows and reducing batch failure rates [187]. Decentralized point-of-care production models, demonstrated by Cellectis, Poseida, and real-world data from India, have successfully produced clinical-grade CAR-T therapies with cost reductions exceeding 50% per patient [188, 189].

In vivo CAR engineering represents an emerging next-generation strategy poised to overcome the manufacturing and logistical bottlenecks of ex vivo CAR-T production. Instead of requiring autologous cell harvesting, genetic modification, and reinfusion, in vivo CAR engineering enables direct programming of immune cells within the patient’s body using targeted delivery platforms. According to Li et al. (2025), current in vivo strategies utilize nanoparticle-based, viral, and bioinstructive scaffold systems to deliver CAR transgenes directly to T cells or other effector immune subsets, thereby streamlining therapy and eliminating complex manufacturing steps. Nanoparticle and viral delivery approaches, in particular, allow localized CAR expression at tumor sites, enhancing safety and minimizing systemic cytokine toxicity. Moreover, implantable scaffolds can serve as microenvironments that promote CAR expansion and persistence in solid tumors such as TNBC. This “off-the-shelf” in vivo CAR engineering paradigm holds potential to significantly reduce costs, shorten turnaround time, and improve accessibility for patients with solid malignancies [190].

To enhance affordability and clinical adoption, economic models emphasizing value-based reimbursement and outcome-linked pricing are increasingly proposed for sustainable CAR-T therapy implementation [184]. Combination strategies pairing dual-target CAR-T cells with checkpoint inhibitors, oncolytic viruses, or ECM-degrading agents show promise in overcoming TME resistance and improving trafficking. Personalized medicine via single-cell antigen profiling and circulating tumor DNA monitoring guides adaptive dual-antigen selection, addressing temporal/spatial heterogeneity and reducing relapse risk [191].

**Fig. 8:**
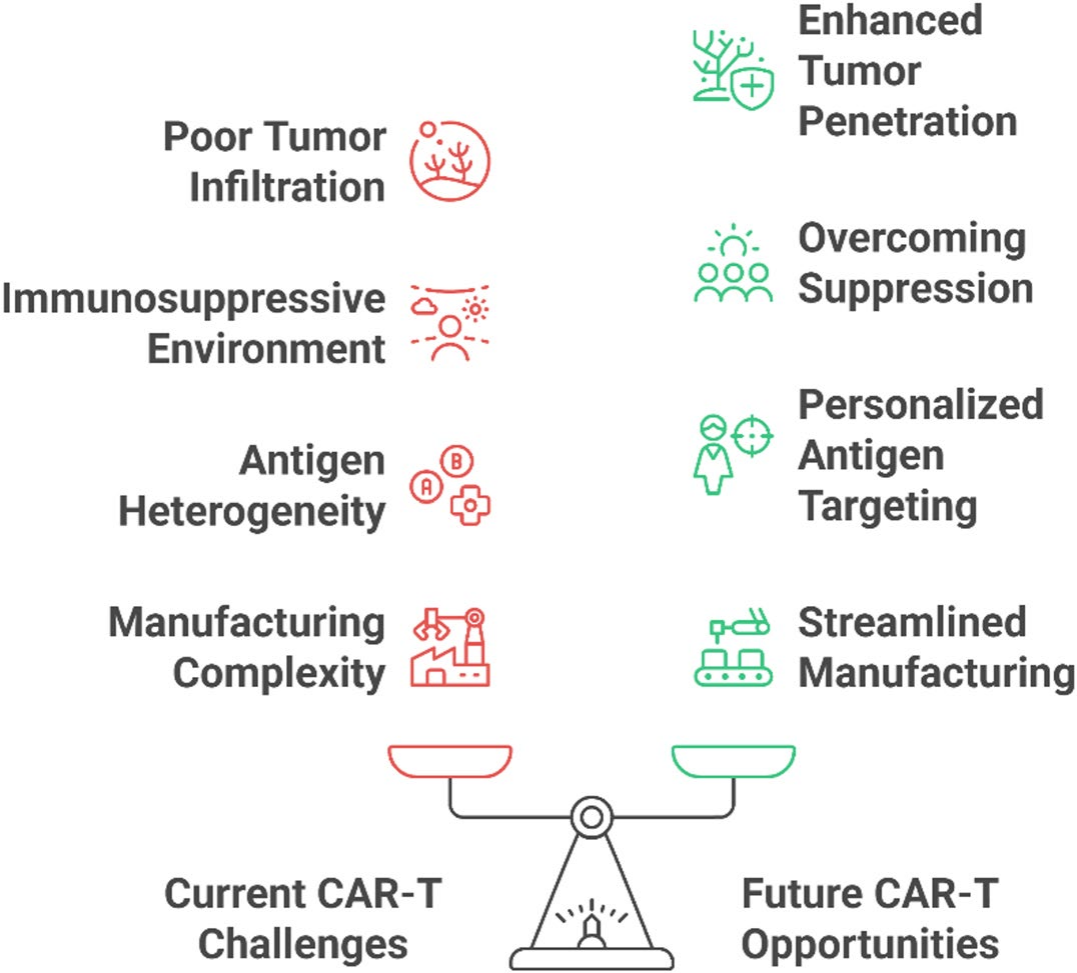
Current challenges and future opportunities for CAR-T-cell therapy in patients with breast cancer

Finally, regulatory and commercialization innovations adaptive trial designs, value-based reimbursement, decentralized manufacturing networks, and harmonized global frameworks can accelerate approval, expand access, and optimize cost-effectiveness, propelling dual-target CAR-T-cell therapy from experimental promise to clinical reality (Fig. 8).

## Conclusion

Dual-targeting CAR-T-cell therapy represents a pivotal evolution in the immunotherapeutic management of breast cancer, offering a strategic framework to overcome the intrinsic challenges of antigenic heterogeneity, immune evasion, and the suppressive TME that have historically constrained single-antigen approaches. By engineering T cells to recognize two distinct tumor-associated antigens through tandem CARs, bicistronic coexpression systems, or programmable logic-gated circuits, this next-generation strategy enhances tumor specificity, mitigates antigen escape, and fine-tunes effector function. The integration of affinity-optimized scFv domains and selective costimulatory motifs (CD28, 4-1BB, or OX40) allows precise modulation of activation thresholds, improving both efficacy and safety in antigen-diverse breast cancer subtypes.

Technological advances in vector engineering, particularly nonviral transposon systems such as *PiggyBac* and *Sleeping Beauty* and AI/ML-driven manufacturing pipelines are accelerating production while reducing costs, enabling scalable and patient-tailored CAR-T manufacturing. Despite these gains, dual targeting introduces unique translational complexities, including reduced transduction efficiency, vector size constraints, and the potential for tonic signaling–induced exhaustion. Clinically, the expanded antigenic scope heightens risks of cytokine release syndrome and off-tumor reactivity, necessitating incorporation of tunable safety mechanisms, such as inducible suicide genes, switchable CARs, and transient mRNA-based platforms. Within the tumor microenvironment, stromal and immunosuppressive barriers such as CAF- and MDSC-derived TGF-β and IL-10 secretion, ECM fibrosis, hypoxia, and metabolic competition continue to impair CAR-T trafficking, persistence, and function. Overcoming these obstacles will likely require rational combination regimens integrating dual-target CAR-T therapy with checkpoint inhibitors (PD-1/CTLA-4 blockade), oncolytic viruses, cytokine modulators (IL-12/15), or ECM-degrading enzymes to remodel the TME and sustain immune activation. The future trajectory of dual-target CAR-T-cell therapy lies in the convergence of synthetic biology, computational immunology, and precision oncology. Adaptive antigen selection guided by single-cell proteogenomic profiling and circulating tumor DNA (ctDNA) monitoring can enable real-time antigen pairing and patient-specific therapy optimization.

Emerging modular CAR platforms such as SynNotch, SUPRA CAR, and universal allogeneic constructs offer programmable logic and controllable activation, expanding clinical applicability while minimizing toxicity. Moreover, decentralized, automated bioreactor systems and AI-driven quality control are redefining manufacturing scalability, shortening vein-to-vein times, and democratizing access across global healthcare systems. To transition dual-target CAR-T-cell therapy from a promising experimental modality to a clinically standardized treatment, concerted efforts must address regulatory harmonization, long-term safety monitoring, and value-based reimbursement frameworks. Collaborative initiatives among academia, biotechnology, and regulatory agencies will be essential to establish robust translational pipelines and equitable distribution models. Continued innovation at the intersection of bioengineering, systems immunology, and regulatory science is poised to transform dual-target CAR-T-cell therapy into a durable, safe, and cost-effective immunotherapy capable of delivering long-term remission and redefining the standard of care in breast cancer.

## Data Availability

All data supporting the results and analysis in this study are fully included within the main article and its supplementary materials. No external datasets were used or generated beyond what is presented in the manuscript.

## Abbreviations

CAR-T: Chimeric antigen receptor T cell
scFv: Single-chain variable fragment
CRS: Cytokine release syndrome
CAF: Cancer-associated fibroblast
MDSC: Myeloid-Derived suppressor cell
ECM: Extracellular Matrix
TME: Tumor microenvironment
TNBC: Triple-negative breast cancer
ICI: Immune checkpoint inhibitor
PD-1: Programmed cell death protein 1
PD-L1: Programmed death-ligand 1
TGF-β: Transforming growth factor
βIL: Interleukin
ICANS: Immune effector cell-associated neurotoxicity syndrome
GVHD: Graft-versus-host disease
TCR: T-cell receptor
HLA: Human Leukocyte antigen
AML: Acute Myeloid Leukemia
AFP: Alpha-Fetoprotein
GPC3: Glypican-3
EGFR: Epidermal growth factor receptor
MUC1: Mucin 1
GD2: Disialoganglioside
GD2 PDX: Patient-Derived Xenograft
EMT: Epithelial–Mesenchymal transition
OS: Overall survival
PFS: Progression-free survival
ORR: Overall response rate
CR: Complete response
PR: Partial response
